# The Chthonomonas calidirosea Genome Is Highly Conserved across Geographic Locations and Distinct Chemical and Microbial Environments in New Zealand's Taupō Volcanic Zone

**DOI:** 10.1128/AEM.00139-16

**Published:** 2016-05-31

**Authors:** Kevin C. Lee, Matthew B. Stott, Peter F. Dunfield, Curtis Huttenhower, Ian R. McDonald, Xochitl C. Morgan

**Affiliations:** a GNS Science, Extremophiles Research Group, Wairakei Research Centre, Taupō, New Zealand; b School of Science, University of Waikato, Hamilton, New Zealand; c Department of Biological Sciences, University of Calgary, Calgary, Alberta, Canada; d Department of Biostatistics, Harvard T. H. Chan School of Public Health, Boston, Massachusetts, USA; e Broad Institute, Cambridge, Massachusetts, USA; f Department of Microbiology and Immunology, University of Otago, Dunedin, New Zealand; University of Buenos Aires

## Abstract

Chthonomonas calidirosea T49^T^ is a low-abundance, carbohydrate-scavenging, and thermophilic soil bacterium with a seemingly disorganized genome. We hypothesized that the C. calidirosea genome would be highly responsive to local selection pressure, resulting in the divergence of its genomic content, genome organization, and carbohydrate utilization phenotype across environments. We tested this hypothesis by sequencing the genomes of four C. calidirosea isolates obtained from four separate geothermal fields in the Taupō Volcanic Zone, New Zealand. For each isolation site, we measured physicochemical attributes and defined the associated microbial community by 16S rRNA gene sequencing. Despite their ecological and geographical isolation, the genome sequences showed low divergence (maximum, 1.17%). Isolate-specific variations included single-nucleotide polymorphisms (SNPs), restriction-modification systems, and mobile elements but few major deletions and no major rearrangements. The 50-fold variation in C. calidirosea relative abundance among the four sites correlated with site environmental characteristics but not with differences in genomic content. Conversely, the carbohydrate utilization profiles of the C. calidirosea isolates corresponded to the inferred isolate phylogenies, which only partially paralleled the geographical relationships among the sample sites. Genomic sequence conservation does not entirely parallel geographic distance, suggesting that stochastic dispersal and localized extinction, which allow for rapid population homogenization with little restriction by geographical barriers, are possible mechanisms of C. calidirosea distribution. This dispersal and extinction mechanism is likely not limited to C. calidirosea but may shape the populations and genomes of many other low-abundance free-living taxa.

**IMPORTANCE** This study compares the genomic sequence variations and metabolisms of four strains of Chthonomonas calidirosea, a rare thermophilic bacterium from the phylum Armatimonadetes. It additionally compares the microbial communities and chemistry of each of the geographically distinct sites from which the four C. calidirosea strains were isolated. C. calidirosea was previously reported to possess a highly disorganized genome, but it was unclear whether this reflected rapid evolution. Here, we show that each isolation site has a distinct chemistry and microbial community, but despite this, the C. calidirosea genome is highly conserved across all isolation sites. Furthermore, genomic sequence differences only partially paralleled geographic distance, suggesting that C. calidirosea genotypes are not primarily determined by adaptive evolution. Instead, the presence of C. calidirosea may be driven by stochastic dispersal and localized extinction. This ecological mechanism may apply to many other low-abundance taxa.

## INTRODUCTION

To date, the poorly characterized phylum Armatimonadetes is described primarily by environmental 16S rRNA marker gene data (1–4) and has only three described type species (5–7), from which two genomes have been sequenced (8, 9). Analysis of these genome sequences shows that Armatimonadetes are most closely related to the candidate lineages FBP and WS1 and to the phylum Chloroflexi (4, 8). Armatimonadetes has three described classes, each represented by a single type strain: Armatimonadia (Armatimonas rosea YO-36^T^) (6), Fimbriimonadia (Fimbriimonas ginsengisoli Gsoil 348^T^) (7), and Chthonomonadetes (Chthonomonas calidirosea T49^T^) (5). The phylum contains at least eight additional class-level phylogenetic lineages without cultivated representatives (3).

Chthonomonas calidirosea T49^T^ is an aerobic and moderately acidophilic thermophile isolated from geothermal soil within the Taupō Volcanic Zone (TVZ), New Zealand (10), an area rich in geothermal systems and surface hydrothermal features. In addition to extracellular polymeric substances, the strain produces extracellular saccharolytic enzymes, which allow it to utilize many diverse carbohydrates, with the exception of crystalline insoluble polymers (e.g., cellulose) (5). Genomic analysis of strain T49^T^ identified a wide range of glycosyl hydrolases and carbohydrate ATP-binding cassette transporters, as well as many extracytoplasmic function sigma factors (8). Based on genomic sequence and physiological characterization, the ecological role of C. calidirosea T49^T^ was proposed to be that of a scavenger, utilizing heterogeneous carbohydrates from degraded biomass within the environment (5, 8). Notably, no C. calidirosea-like phylotypes have been reported from outside the TVZ (3, 11, 12). The most similar phylotypes (based on pairwise comparison, GenBank accession numbers KM102610 and KM102602 have 93% 16S rRNA gene sequence identity to strain T49^T^) originated from volcanic soils at Paricutin Volcano, Mexico (see also the supplemental material).

One remarkable feature of strain T49^T^ is its apparent genomic disorganization. Many functionally related genes that are typically organized into operons in other bacterial genomes (e.g., histidine, tryptophan, and purine biosynthesis) are instead individually distributed throughout the C. calidirosea genome. This genomic disorganization complicates metabolic pathway predictions, although the abundant sigma factors observed in the genome provide a potential mechanism for gene regulation (8, 13). The high dispersal of functionally related genes observed in C. calidirosea appears to be uncommon. High genomic disorganization is observed in some cyanobacterial species (14–16), as well as in the Armatimonadetes species F. ginsengisoli (1) (see Fig. S1 in the supplemental material). Among the cyanobacteria, Prochlorococcus genomes are highly plastic and frequently reorganized by bacteriophages in response to their environment (17). Prochlorococcus genomes often display shorter and more dispersed operons than are found in other bacterial genomes (14, 18, 19). We therefore hypothesized that the nonoperonic genome organization of strain T49^T^ would likewise be highly plastic and responsive to the selection pressure of the immediate environment, and this plasticity would be phenotypically reflected in traits such as the repertoire of carbohydrate utilization in distinct environments.

In order to assess the degree of genomic flux within the C. calidirosea genome in response to environmental selection pressure, we compared the genome sequences of strain T49^T^ to those of three additional C. calidirosea isolates cultured from geographically isolated sites, averaging 46.2 km from the site of strain T49^T^ isolation. In addition, we collected environmental geochemistry data and defined the microbial community structures by assessing the 16S rRNA gene sequence diversity for each sample site. With these data, we evaluated the relationships among geographic distance, geochemistry, community diversity, and genomic sequence variation in C. calidirosea.

## MATERIALS AND METHODS

### Cultivation of Chthonomonas calidirosea isolates.

**FIG 1:**
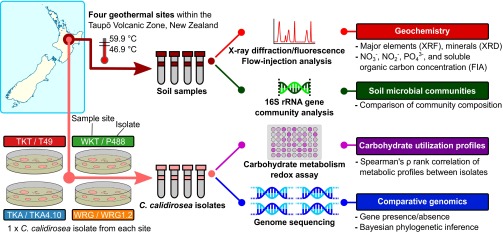
Overview of data collection and analysis. Soil samples were collected from four geothermal sites across the Taupō Volcanic Zone (TVZ): TKT, WKT, WRG, and TKA. C. calidirosea was isolated from each site. Soil mineral content was measured by X-ray diffraction/X-ray fluorescence (XRD/XRF) and flow injection analysis (FIA). Extracted soil DNA was subjected to 16S rRNA gene sequence analysis to determine microbial community composition. C. calidirosea isolates were metabolically profiled, and their genomes were sequenced. For each method, corresponding data types and/or analytical results are listed to the right. The sample sites and the C. calidirosea isolates are color coded.

Based on 16S rRNA gene clone libraries constructed from diverse sites within the TVZ (data not shown), four geographically separated geothermal systems (Tikitere [TKT], November 2006; Waikite [WKT], July 2006; Wairakei [WRG], April 2009; and Te Kopia [TKA], September 2010) containing Armatimonadetes/candidate division OP10 were chosen for this study. Four pure cultures of C. calidirosea (T49^T^, P488, WRG1.2, and TKA4.10) were isolated from these sites under cultivation conditions previously described for oligotrophic carbohydrate-utilizing thermophiles (10) (Fig. 1). Strain T49^T^ was physiologically characterized in a previous study (5).

### Genome sequencing, assembly, and quality assessment. (i) Sequencing of C. calidirosea isolate genomes.

After cultivation, genomic DNA was extracted from isolates P488, WRG1.2, and TKA4.10 using a previously described phenol-chloroform method (8). The four genomes were sequenced via different technologies (Table 1). Strain T49^T^ was sequenced in a previous study (8). For isolates P488 and WRG1.2, genomic DNA libraries were constructed using the Nextera XT DNA sample preparation kit (Illumina, Inc., San Diego, CA, USA), according to the manufacturer's protocol, and 2 × 150-bp paired-end sequencing was performed using a 300-cycle sequencing kit (version 1.0) on an Illumina MiSeq. For isolate TKA4.10, a genomic DNA library was constructed using the Ion Xpress Plus fragment library kit with 100 ng of input DNA. The beads were prepared using the Ion One Touch 200-bp version 2 DL kit, and sequencing was performed with the Ion PGM 300-bp sequencing kit on an Ion Torrent (Thermo Fisher Scientific, Inc., Waltham, MA, USA).

**TABLE 1:** Genome sequencing statistics of the four C. calidirosea isolates

Isolate	Source	Sequencing technology	No. of reads assembled	Coverage (×)	Genome size (bp)	G+C content (%)	No. of assembled protein-coding genes	No. of 5S, 16S, 23S rRNA genes	No. of tRNA genes
T49^T^	Hell's Gate, Tikitere, North Island, NZ	454 Titanium + Sanger	171,649	20	3,437,861	54.41	2,877	1, 2, 1	46
P488	Waikite Valley, North Island, NZ	Illumina MiSeq	2,533,602	88	3,438,278	53.93	2,876	1, 2, 1	46
WRG1.2	Wairakei, North Island, NZ	Illumina MiSeq	3,272,279	112	3,438,088	53.57	2,855	1, 2, 1	46
TKA4.10	Te Kopia, North Island, NZ	Ion Torrent	954,245	39	3,437,861	54.41	2,885	1, 2, 1	46

### (ii) Assembly and annotation of isolate genomes.

MIRA version 4.0rc3 (20) was used to assemble the genomes of P488, WRG1.2, and TKA4.10. The strain T49^T^ genome (8) was used as a reference during assembly. This resulted in a single scaffold for each genome, in which ambiguous regions were indicated with repeating Ns (IUPAC notation). The resulting assemblies were aligned using progressiveMauve (21). Assembly coverage (Table 1) was calculated from contigs of ≥5,000 bp to avoid inflation of the figures. Gene prediction and annotation of the genomes were performed using the Integrated Microbial Genome-Expert Review (IMG-ER) pipeline (22). Further details regarding sequencing, assembly, and quality control of genome assemblies are described in Fig. S2 and S3 in the supplemental material.

### Genome comparison and phylogenetic analysis. (i) Identification of gene homologs across C. calidirosea isolates.

To determine whether annotated genes were conserved between strains, we calculated the read coverage for each gene in each strain using Bowtie2 (23) and BEDtools (24). The complete code for calculating gene coverage is available at http://bitbucket.org/biobakery/chthonomonas. The identification of conserved homologs between genomes with UCLUST (25) and the identification of putative genes in unmapped reads are described in the supplemental material and are summarized in Fig. S4.

### (ii) Determination of isolate phylogeny.

The PhyloPhlAn gene collection of 400 ubiquitous and phylogenetically informative genes (26) was used for phylogenetic analysis of the isolates. The C. calidirosea genomes contained 327 of these genes (see Table S1 in the supplemental material). Due to the high similarity between the isolates, we conducted Bayesian phylogeny inference using nucleic acid sequences rather than the filtered protein sequences used by PhyloPhlAn; this increased the isolate-level phylogenetic resolution and enabled the assessment of nucleic acid sequence divergence (substitutions per site). Each gene was first separately aligned (aligning the four homologs of the gene from the four isolates) using MUSCLE (27) without the removal of invariant sites. The multiple alignments were then concatenated and used for phylogenetic inference with MrBayes (28). The genes were divided into partitions and were unlinked so that each partition had its own set of parameters for estimation during the Markov chain Monte Carlo (MCMC) process. Each partition used the general time-reversible substitution model, with the heterogeneity rate determined by the invariable site plus gamma distribution (GTR + I + Γ). The model was selected using jModelTest (29) with the Akaike information criterion (AIC). Two MCMC runs were conducted. Each MCMC process ran for 140 million generations, sampling every 1,000 generations, with 35 million (25%) generations as burn-in. MCMC performance diagnostics were conducted via Tracer (http://tree.bio.ed.ac.uk/software/tracer/).

### (iii) Genomic rearrangement comparison with other thermophiles.

We compared the degree of genomic sequence rearrangement observed in C. calidirosea to that observed in other thermophilic species isolated from defined geographic locations. The genomic sequences of Sulfolobus islandicus (strains Y.N.15.51 and Y.G.57.14) and Thermus thermophilus (strains HB-8, HB-27, and JL-18) were downloaded from NCBI GenBank, and the genomic rearrangement of each species was assessed by aligning all of its strains (including those of the C. calidirosea isolates) using progressiveMauve (21). PhyloPhlAn and MUSCLE were used to select and align conserved genes within the genome sequence of each compared species (i.e., C. calidirosea, T. thermophilus, and S. islandicus). Sequence identities/dissimiliarities were calculated from the concatenated aligned gene sequences with SIAS (http://imed.med.ucm.es/Tools/sias.html). Due to an incomplete assembly (>100 contigs), T. thermophilus strain ATCC 33923 could not be analyzed for genomic sequence rearrangement, but the genome sequence was used for assessment of the divergence of conserved genes. Further details are described in the supplemental material and are summarized in Fig. S5.

### Characterization of C. calidirosea isolate metabolisms with Biolog phenotype microarrays.

The substrate utilization phenotypes of the four C. calidirosea isolates were compared using Biolog phenotype microarrays (Biolog, Inc., Hayward, CA, USA). All isolates were maintained using 4.5NZS solid medium (10). Colonies were collected from the medium after 1 week of incubation at 60°C and were used to inoculate 250 ml of 4.5NZS liquid medium (pH 5.5), and 3 g/liter maltose and 0.2 g/liter Casamino Acids were added in 500-ml containers under an air headspace. The liquid cultures were incubated at 60°C for ∼40 h. Cells from 50 ml of culture were pelleted by centrifugation (5,190 × *g* for 15 min) and washed with sterile water three times prior to resuspension in a maltose-free 4.5NZS liquid medium at an optical density at 600 nm (OD_600_) of 0.6 to 0.8. A 100-μl aliquot of the resuspended culture and 5 μl (5 g/liter) of MTT [3-(4,5-dimethylthiazol-2-yl)-2,5-diphenyltetrazolium bromide] were added to each of the 96 wells on a Biolog PM1 plate (Biolog, Inc.). The plates were sealed and incubated in darkness at 60°C for ∼23 h. After incubation, 900 μl of dimethyl sulfoxide was added to each well (30), and the OD_540_ was recorded. Each isolate was tested twice, using a separate cultivation batch for each experimental replicate. Mean responses were calculated from the corresponding wells of each isolate, and tie-corrected Spearman rank-order correlations between isolates were calculated with the R function cor.test (31) (see Fig. S6 and Table S9 in the supplemental material).

### Analysis of soil chemistry and mineral content.

Soil temperature was measured on site using a Fluke 50S thermocouple. Sample pH was measured in the laboratory at 25°C by mixing 1 g of the sample in 10 ml of deionized water. Soil moisture content and major oxides were analyzed using X-ray fluorescence (XRF). Major oxides were detected by XRF with borate fusion of the samples. The total soil carbon content was measured by mixing weighed soil aliquots with 25 ml of 0.05 M K_2_SO_4_ and filtering to extract total soluble carbon. Total soluble carbon was then measured by combusting the samples and measuring the CO_2_ evolution using a multi N/C 3100 analyzer (Analytik Jena AG, Jena, Germany). The inorganic carbon content of the sample was measured separately by quantifying the CO_2_ generated by the acidification of an aliquot of the original sample (32). Mineral content was determined by X-ray diffraction (XRD) (Philip X'Pert Pro with Co Kα radiation source) with the software X'Pert HighScore (Spectris plc, England) and Siroquant (Sietronics Pty. Ltd., Canberra, Australia) (see Fig. S7 in the supplemental material). Soil nitrate, nitrite, ammonia, and dissolved reactive phosphate levels were determined by flow injection analysis (FIA) (Hach Company, Loveland, CO, USA). Analytes were extracted by mixing 1 g of soil sample with 50 ml of 1 M KCl. The mixture was centrifuged, and the resulting supernatant was passed through a 0.2-μm-pore-size filter prior to analysis.

### Community 16S rRNA gene-targeted sequencing and bioinformatic processing.

Total DNA was then extracted from each soil sample. Prior to DNA extraction, each soil sample from each site was mixed with 500 μl of a 50-g/liter sterile skim milk solution (Becton, Dickinson and Company, NJ, USA) to reduce DNA binding to clay. DNA was then extracted with a NucleoSpin soil DNA kit (Macherey-Nagel GmbH & Co. KG, Düren, Germany) according to the manufacturer's instructions. The V4 hypervariable region of the 16S rRNA gene was amplified by PCR and sequenced using the Ion Torrent platform, according to standardized methods, as described in detail in the supplemental material. Quality control was performed with UPARSE (33), as described in detail in the supplemental material, resulting in a mean of ∼20,700 sequences per sample. These sequences were clustered *de novo* into operational taxonomic units (OTUs), with a minimum identity of 97%, and the representative sequences were taxonomically assigned using the UCLUST (25) consensus taxonomy assigner in QIIME 1.8.0 (34). Greengenes release 13_8 (35) was used as the reference taxonomic database. The OTU table was rarefied to 17,941 sequences per sample to remove sample heterogeneity; rarefaction analysis indicated that the sequencing depth was sufficient to identify the majority of OTUs within each community (see Fig. S8 in the supplemental material). QIIME (34) was used for β-diversity calculations and downstream analyses; β-diversity was measured by Bray-Curtis dissimilarity and by weighted and unweighted UniFrac indices. A weighted-UniFrac hierarchical clustering tree was constructed using the unweighted pair group method using average linkages (UPGMA). Jackknife analysis with the UPGMA tree was conducted with 200 repetitions and 12,000 sequences per sample. PyNAST alignment (36) and FastTree (37) were used to generate phylogenetic trees.

### Accession numbers.

The genome sequence and assembly data of the C. calidirosea isolates were deposited at EMBL/GenBank with the following accession numbers: PRJEB1573 (strain T49^T^), PRJEB4907 (isolate P488), PRJEB4936 (isolate WRG1.2), and PRJEB4937 (isolate TKA4.10). Community sequencing data were deposited under accession number PRJEB13454.

## RESULTS

The aim of this research was to understand how the ecosystem shapes the evolution of the C. calidirosea genome and phenotype. To connect genome dynamics with environmental factors and observed phenotypes, we isolated four C. calidirosea strains from four geographically distinct geothermal soil environments. For each site, we determined the soil chemistry and microbial community composition by 16S rRNA gene sequence analysis. We sequenced each isolate genome and performed nucleic acid sequence divergence assessment, phylogenetic inference, and gene content and organization analysis. In addition, we characterized the metabolic profile of each isolate (Fig. 1).

### Sample sites show major differences in clay content and hydrothermal activity.

The four sample sites were distributed along the TVZ (see Fig. S9 in the supplemental material), with a maximum linear distance of 67.4 km and minimum of 12.0 km between sites. All sample sites were geothermally affected soils. Soil pH ranged from 3.5 to 4.8, and the temperature ranged from 46.9 to 59.9°C. We characterized the mineral compositions of the sample sites using semiquantitative XRD and XRF analyses (see Fig. S7 and Table S2 in the supplemental material). The soil minerals were primarily quartz, silica, and clay. Clay minerals are sensitive to hydrothermal alteration. As shown in Fig. S7, the clay types at sites TKA and TKT were almost entirely primary clay minerals (e.g., magnetite, biotite, pyroxene, and plagioclase), indicating that these ecosystems were either newly affected or weakly affected by hydrothermal activity. In contrast, sites WKT and WRG had mostly secondary clay minerals (e.g., alunite-kaolinite and montmorillonite), which are formed from the reaction of rock with acidic steam-heated water over an extended period of time (38–40) (Table 2). WKT had the highest clay content, followed by WRG and TKT, while TKA was mostly quartz (86% [wt/wt]) (see Fig. S7). WKT was also much richer in soluble organic carbon (SOC) (2.33 mg/g of soil) than the other sites (range, 1.06 to 1.59 mg/g of soil) (Table 2). This SOC concentration was comparable to that of ambient-temperature soil environments, such as the nutrient-rich crop field soil (6.1 mg/g of soil) (41) and nutrient-poor pine plantation forest soil (0.08 to 0.17 mg/g of soil) (42), indicating that organic carbon is available at the sample sites.

**TABLE 2:** General physicochemistry of soil samples

Sample site (isolate)	Temp (°C)	pH	Moisture (%)	SOC (mg/g of soil)^*a*^	Ammonia (mg/g of soil)	Nitrate (mg/g of soil)	Nitrite (mg/g of soil)	Dissolved reactive phosphorus (mg/g of soil)^*b*^
TKT (T49^T^)	52.5	4.3	28.0	1.47	145.0	2	0.15	BDL
WKT (P488)	50.7	4.5	59.9	2.33	105.0	6	0.35	BDL
WRG (WRG1.2)	46.9	4.8	37.1	1.06	28.5	2	0.40	BDL
TKA (TKA4.10)	59.9	3.5	28.8	1.59	23.5	1	1.50	BDL

a SOC, soluble organic carbon.

b BDL, below detection limit.

### C. calidirosea is a low-abundance taxon in diverse communities dominated by Crenarchaeota and Thaumarchaeota.

We determined the relative abundance of C. calidirosea and other taxa at each sample site by community 16S rRNA gene sequencing. We observed a total of 190 operational taxonomic units (OTUs) among the four sites (see Table S3 in the supplemental material). A single OTU representing the genus Chthonomonas (OTU_085, with 99% sequence identity to strain T49^T^) was detected at very low relative abundance (ranging from 6 × 10^−5^ [TKA] to 3 × 10^−3^ [WKT]) in all four communities (see Table S4 in the supplemental material). Although the short read length and error rate of ion semiconductor sequencing (Ion Torrent) cannot support high-resolution comparison, these data confirm the detection of Chthonomonas with limited phylogenetic diversity (within the 97% OTU clustering criterion). The C. calidirosea OTU was most abundant in the more nutrient-rich soil at WKT, which had higher soluble organic carbon content, nitrate concentration, and overall clay mineral content (Table 2; see also Fig. S7 and Tables S3 and S4 in the supplemental material). In contrast, C. calidirosea was less abundant in sites consisting mainly of fresh hydrothermal deposits lacking in soluble organic carbon but rich in quartz and amorphous silica, as represented by the SiO_2_ concentration (see Table S2 in the supplemental material). Clay and organic matter increase the buffering capacity of soil (43), which may facilitate the survival of C. calidirosea, a species with a very limited pH range for growth (5, 8).

**FIG 2:**
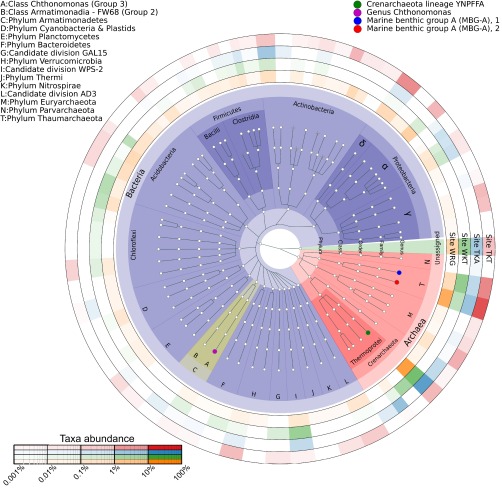
Microbial communities of the sample sites. Each ring on the outside of the tree corresponds to a sample site, and the color intensity at each position on the ring indicates the relative abundance of the corresponding taxon at that sample site. The color intensity has been scaled nonlinearly to emphasize low-abundance taxa by transforming the relative abundance with the exponent (⅓). Cross symbols at the terminal nodes indicate representative reads with only higher-level taxon assignments and may contain multiple genera. The bacterial phylum Armatimonadetes is highlighted in yellow, and the member genus Chthonomonas of class Chthonomonadetes (labeled “A”) is colored in magenta. The relative abundance of Chthonomonas-related OTUs at each of the four sample sites was as follows: WRG, 4.08 × 10^−4^; WKT, 3.02 × 10^−3^; TKA, 5.6 × 10^−5^; and TKT, 1.17 × 10^−4^.

We observed only 21 archaeal OTUs, but these OTUs comprised the majority of the community at all four sites (56% [WKT] to 75% [TKA]). Five archaeal OTUs were present at all sites. Nearly half of observed archaeal OTUs (10/21) were Euryarchaeota, all belonging to the thermophilic acidophilic class Thermoplasmata (44). However, these represented <5% of the total sequence reads from any community. In contrast, the Crenarchaeota and Thaumarchaeota comprised the majority of reads from each community (Fig. 2). The Crenarchaeota, which were mainly Thermoprotei (nine OTUs [39 to 58% of total sequence reads]) (45), dominated all communities except TKT. The deep-branching MBG-A group (45–48) (12 OTUs [13 to 28% of total sequence reads]) dominated the TKT community (57% of OTUs). In this study, the class Thermoprotei was predominantly represented by the lineage YNPFFA, which is also associated with geothermal features at Yellowstone National Park, USA (49). Unfortunately, the lack of cultivated representatives within these Crenarchaeota and Thaumarchaeota lineages impedes any attempt to infer their ecological roles.

The four communities were distinctly different at a finer taxonomic resolution. Only 25 of the 128 total bacterial OTUs were observed at all four sites, and none of these were highly abundant (see Table S3 in the supplemental material). These taxa included Sulfobacillaceae, Rhodospirillales, and Acidimicrobiaceae. Based on their affiliations to characterized type strains, we speculate that these bacteria are moderately acidophilic, chemolithotrophic, and aerobic, and they grow via mechanisms, such as iron and sulfur oxidation (Sulfobacillaceae [50]), iron oxidation (Acidimicrobiaceae [51]), or methanotrophy (“Candidatus Methylacidiphilum” [52]). In contrast, many predominant OTUs were detected in only one or two sites. For example, an archaeal OTU from clade SK322 (OTU_0003) comprised 10.3% of the total reads from WKT but was not detected at TKT, while an Acinetobacter OTU (OTU_158) comprised 8.5% of the total reads from WKT but was undetected at the other sites (see Table S3). These observations indicate that although the four C. calidirosea-hosting geothermal systems were broadly similar (in pH, temperature, and hydrothermally affected clays), they did not support similar microbial community compositions. This suggests that other ecological or stochastic differences determine the abundance of Chthonomonas spp. within these ecosystems.

### C. calidirosea genomic content and organization are highly conserved between isolates.

The genomes of C. calidirosea isolates P488, WRG1.2, and TKA4.10 were sequenced using the Illumina MiSeq or Ion Torrent platform (Table 1) and assembled using the previously sequenced T49^T^ genome (8) as a template. MIRA (20) and SPAdes (53) produced very similar assemblies (see the supplemental material), indicating little bias due to assembler choice. The length and G+C content of the TKA4.10 genome were identical to those of T49^T^; this was potentially influenced by the inefficient assembly of single-ended Ion Torrent reads beyond reference contig boundaries. Isolates P488 and WRG1.2 had slightly longer genome assemblies and lower G+C content than T49^T^ and TKA4.10 (Table 1).

**FIG 3:**
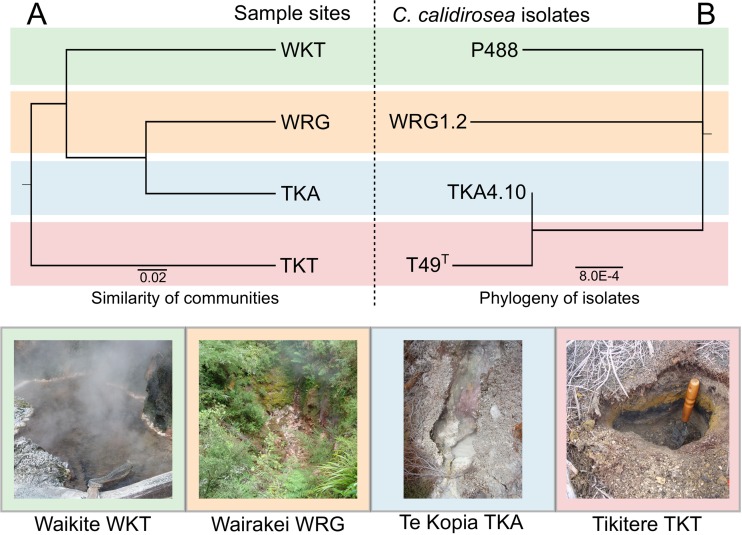
Similarity between C. calidirosea isolate phylogeny and sample site communities. (A) Sample site community similarity is shown by a jackknifed-UPGMA tree, created using weighted UniFrac distance. The community tree has high bootstrap support values (75 to 100%) for all internal nodes. (B) Phylogenetic relationships among the four *C*. calidirosea isolates, shown by an unrooted Bayesian nucleic acid phylogenetic tree of 327 concatenated conserved genes shared by the four isolates. The branching of the phylogenetic tree is supported by 100% posterior probability, with the branch length indicating the expected nucleotide changes per site. Photos of the sample sites are shown below the trees.

We determined the phylogeny of the strains by aligning the concatenated nucleic acid sequences of 327 phylogenetically informative genes (26) (see Table S1 in the supplemental material) and building a phylogenetic tree using Bayesian inference (Fig. 3). The pairwise sequence identity of these genes was very high (average, 99.2%) between the isolates. Based on these data, strain T49^T^ shares the highest similarity with TKA, followed by P488 and WRG1.2. The posterior probability support for this unrooted tree topology was >99%. Within this tree, TKA4.10 showed a very short branch length from the internal node shared with T49^T^. WRG1.2 had the longest terminal branch length. This is in slight disagreement with strain-level conservation of the 16S rRNA gene sequences; each genome had two copies of the 16S rRNA gene, and all are identical except in strain P488, in which the two copies differ by 1 base.

While the four C. calidirosea genome sequences showed a high degree of synteny in most genomic regions (see Fig. S2 and S5 in the supplemental material), a previous study identified two putative horizontally transferred regions, A (between the loci CCALI_00447 and CCALI_00449) and B (between the loci CCALI_00804 and CCALI_00807) (8) (see Fig. S10 in the supplemental material). These two regions were conserved between isolates T49^T^ and TKA4.10 but are mostly absent in isolate WRG1.2. Isolate P488 contained only a small deletion in region B, and the sequences in both regions were slightly less conserved. The variability in these regions is consistent with our inferred strain phylogeny (Fig. 3); there is low divergence between T49^T^ and TKA4.10, more divergence in P488 (e.g., deletions, truncations, and frameshift mutations in the loci CCALI_00449 and CCALI_00805), and the regions were either never acquired or deleted from WRG1.2.

Finally, we compared our Bayesian-inferred strain phylogeny to the structure of the microbial community from each sample site, as measured by β-diversity (Fig. 3; see also Table S5 in the supplemental material). When communities were compared by metrics that consider OTU abundance (e.g., weighted UniFrac and Bray-Curtis dissimilarity), sites WRG and TKA had the most similar communities, followed by WKT and then TKT. In contrast, isolates TKA4.10 and T49^T^ are phylogenetically the most similar, indicating that the differences in genome sequence phylogeny of C. calidirosea isolates are not reflected by differences in the community composition of their respective isolation sites.

### Functionally annotated isolate-variant genes are primarily DNA-interacting enzymes.

We next assessed the similarity of genetic contents between the genomic assemblies by aligning the reads from each isolate to the genes from all four isolates and then assessing the coverage of each gene in each strain (see the supplemental material). Most gene homologs (∼2,600) were present in all four C. calidirosea isolates, but we identified 769 putative isolate-variant genes, many of which encoded hypothetical proteins or domains of unknown function (see Table S6 in the supplemental material). There were a total of 50 apparently isolate-unique genes among the four isolates; the majority (31/50) of these were hypothetical proteins. Of the remainder, the most common functions were related to DNA modification (e.g., restriction enzymes and methylation).

Finally, we examined the genome sequences of the isolates for genes that were not assembled into scaffolds due to the use of strain T49^T^ as a reference during assembly (i.e., genes absent from the T49^T^ genome sequence but present in other isolates). We performed a *de novo* assembly of the unassembled reads for each genome and then used a blastn search (NCBI nr database) to search these contigs for genes. In this manner, we identified 14 additional putative functionally annotated genes absent from strain T49^T^ (see Table S7 in the supplemental material).

### The C. calidirosea genome is more highly conserved across geographic distance than Sulfolobus islandicus or Thermus thermophilus.

The four C. calidirosea isolates exhibited very low genomic divergence across a substantial geographic distance (see Fig. S9 in the supplemental material). We compared the nucleic acid sequence divergence (0.33% to 1.17%) (Table 3) and genomic sequence rearrangement (see Fig. S5 in the supplemental material) of the C. calidirosea isolates with two other well-studied groups of thermophilic microorganisms: Thermus thermophilus and Sulfolobus islandicus (54, 55). The sets of both T. thermophilus and S. islandicus strains were isolated across a geographic scale comparable to that of the C. calidirosea cohort (see the supplemental material). Relative to C. calidirosea, nucleic acid sequence divergence was more than double (2.45%) in S. islandicus and more than triple (3.18% to 7.49%) in T. thermophilus (see Table S8 in the supplemental material). Both of these species also exhibited greater genomic sequence rearrangement across strains than was evident in any two C. calidirosea genome sequences (see Fig. S5).

**TABLE 3:** Conservation between C. calidirosea isolate genomes across an alignment of 327 phylogenetically informative genes (26)^*a*^

Isolate	Unmapped sequence size (bp)	Total no. (avg [%]) of base differences for:		
		T49^T^	P488	WRG1.2
T49^T^	488,944			
P488	488,769	5,039 (1.03)		
WRG1.2	488,350	5,736 (1.17)	3,769 (0.77)	
TKA4.10	488,323	1,627 (0.33)	3,815 (0.78)	4,518 (0.92)

a The total length of the multiple-sequence alignment was 489,735 bp. The average base differences are per 100 bases.

### Substrate utilization is conserved across C. calidirosea isolates, but the relative activities are isolate specific.

Strain T49^T^ was physiologically and genomically characterized as a scavenger capable of utilizing a wide range of carbohydrates (5, 8). To assess the extent to which each strain had physiologically adapted and specialized to its environment, we conducted carbohydrate utilization redox assays with Biolog PM1 carbon source microtiter plates. The plates contained 95 substrates, including mono- and disaccharides, amino acids, sugar alcohols, nucleosides, organic acids, and other potential energy sources. All isolates had similar substrate utilization profiles, with the highest redox response to hemicellulosic sugars (e.g., xylose and mannose) and negligible responses to amino acids as a sole carbon and energy source. However, the relative intensity of substrate response was isolate specific and reproducible. We assessed isolate substrate response similarity by calculating the Spearman rank-order correlation coefficients between the carbon utilization profiles (see Fig. S6 and Table S9 in the supplemental material). The most similar utilization profiles were from P488 and WRG1.2 (ρ = 0.943), while the least similar were T49^T^ and WRG1.2 (ρ = 0.768). These substrate utilization differences do not appear to be due to variation in genes related to carbon metabolism. They may, therefore, be due to differences in regulatory genes or polymorphisms in functional genes, such as enzymes or transporters.

## DISCUSSION

### Role of C. calidirosea as a heterotrophic scavenger in microbial communities.

Our 16S rRNA gene analysis of the four C. calidirosea-associated communities revealed that Crenarchaeota and Thaumarchaeota dominated all communities, with diverse bacterial species as minor components. The variation in site community composition is likely influenced by their varied degrees of hydrothermal activity and different geochemistries. A previous culture-based study of the sample site WKT (10) resulted in the isolation of several bacterial taxa (including C. calidirosea) but no archaeal species. The dominant archaeal lineages detected in this study are only distantly related to any characterized isolates. However, based on the metabolic capabilities of described strains within related lineages (e.g., Sulfolobales, Nitrososphaerales, and Nitrosopumilales), lithoautotrophic lifestyles would seem likely (56–59). We therefore speculate that the dominant crenarchaeotal and thaumarchaeal phylotypes may occupy the niche of autotrophic primary producers, supporting diverse, yet low-abundance, chemoheterotrophic bacterial species, like C. calidirosea. The scavenger phenotype of C. calidirosea may be well suited to persist in an ecosystem with minimal or inconsistent saccharide sources (5, 8). A recent study of bacterial communities in a Thailand hot spring also indicated a potential relationship between Armatimonadetes and autotrophs. This survey detected abundant Armatimonadetes and Chloroflexi OTUs associated with cyanobacterial mats (60). Pairwise discontiguous megaBLAST analyses between the Thailand hot springs Armatimonadetes OTUs and C. calidirosea showed that the Thailand OTUs had low sequence similarities (75 to 84%) to the type strains of the three Armatimonadetes classes (see Table S10 in the supplemental material).

### Low genomic diversity in the face of geographical isolation.

Although the four C. calidirosea isolates were cultured from geographically distant sites across the TVZ, their genome sequences were highly conserved, particularly compared to other thermophilic microorganisms recovered across similar geographical scales (see Fig. S5 and Table S8 in the supplemental material). Phylogeny inferred from single-nucleotide polymorphisms (SNPs) and horizontal gene transfer indicated a close relationship between all isolates, particularly between T49^T^ and TKA4.10. Gene presence/absence analysis identified relatively few variant genes (see Tables S6 and S7 in the supplemental material), such as restriction-modification systems. These genes are known to be rapidly evolving (61) and mobile (62), which may explain their presence in an otherwise largely conserved pangenome. Physiologically, all four C. calidirosea isolates had similar carbohydrate metabolism. Future comparative transcriptomic and proteomic analyses of the isolates may shed light on subtle underlying regulatory processes resulting in the variations of phenotypic response within these highly conserved genome sequences.

### Potential mechanisms underpinning genomic conservation across geographic distance.

The divergence between genome sequences of C. calidirosea isolates consisted primarily of SNPs, which may accumulate by genetic drift during geographic isolation. However, the phylogenetic distances of the isolate genome sequences do not reflect the geographic relationships of the sample sites (see Fig. S9 in the supplemental material). The reason for the low genomic sequence divergence between the C. calidirosea isolates despite their geographical and ecological isolation is not immediately clear. One possible explanation for the high genomic sequence conservation is a relatively recent sympatric determination of species of C. calidirosea from a mesophilic relative, leading to its occupation of a new niche in geothermal environments. A review of 16S rRNA gene data sets available in the NCBI and EMP databases indicates that C. calidirosea can presently be considered endemic to New Zealand, with no genus-level phylotypes detected elsewhere (see the supplemental material). A recent review of the environmental distribution of Armatimonadetes phylotypes indicated that temperate soil is the most dominant environment for the phylum (3). Of 39 phylotypes identified representing the class Chthonomonadetes (group 2), only two phylogenetically distant clones were associated with thermophilic environments. In contrast, group 10 (A and B) consists entirely of phylotypes from geographically disparate geothermal environments. Thus, the occasional occurrence of thermophily in the Chthonomonadetes may indicate either recently acquired thermophilic adaptation or sampling bias. The identification of more closely related phylotypes in the future will help clarify the evolutionary history and environmental distribution of Chthonomonas.

Although this hypothesis may explain the low diversity from an evolution and sampling perspective, it is inadequate to explain the very high similarity in the genome sequences from genetic drift, unless the dispersal of C. calidirosea was relatively recent. The high similarity between strain T49^T^ and isolate TKA4.10 suggests recent dispersal between the two sites. A recent study found that thermophilic microbes can be globally dispersed via aeolian transport but are selected by their environments (63). In dynamic geothermal environments, taxa with very low abundance, such as C. calidirosea, may become locally extinct but subsequently recolonized from another site, resulting in high genetic similarity between two populations. Over a sufficiently rapid timespan of extinction and colonization, the genome sequences would reflect stochastic aeolian dispersal rather than adaptive responses to selection pressures of specific environments. In contrast, an abundant species would be less sensitive to the stochastic process of localized extinction and repeated seeding and thus more likely to present a clearer relationship between geography and phylogenetic distance. We believe the low diversity of C. calidirosea genome sequences potentially reflects these scenarios. While surface and groundwater water flow presents an alternative mechanism of dispersal between sites, this is unlikely due to the arrangement of river catchments and aquifers flowing away from individual sites (see Fig. S9). The environmental persistence of C. calidirosea is further restricted by its inability to sporulate and by its fastidious growth requirements (limited pH and temperature growth ranges). Thus, rapid dispersal between suitable habitats is a possible mechanism for maintenance of local populations and genome consistency.

Based on analysis of the genome sequences of C. calidirosea isolates, we reject our initial hypothesis that C. calidirosea genomic disorganization reflects plasticity and niche-specific adaptation. Instead, the genome sequence is highly conserved and shows a small degree of genetic drift rather than response to different ecological selection pressures at different sample sites. To date, C. calidirosea has been detected only within New Zealand's TVZ, and its global distribution is currently unknown. If additional isolates can be cultured from greater geographic distances (such as the fumarolic/volcanic-associated phylotypes of Paricutin Volcano, Mexico, which share 93% 16S rRNA gene sequence identity), they will continue to improve our understanding of how the C. calidirosea genome is shaped by genetic drift. Rather than possessing a dynamic and highly plastic genome, the conservation of gene content and order (synteny) of C. calidirosea may be comparable to those of prokaryotes such as Haloquadratum walsbyi (64) and members of the SAR11 (Pelagibacterales) clade (65), which are known for high genomic conservation.

### Conclusion.

In order to investigate the relationship between the C. calidirosea genome and its environment, we performed comparative genomics and determined the physiology of isolates obtained from four distinct sites. These data were integrated with microbial community analyses and environmental geochemistry data for the respective sites. We have shown that C. calidirosea, a thermophilic non-spore-forming bacterium, exhibits detectable, albeit minor, genome sequence differences across a geographic distance. We propose that this bacterium is capable of dispersal across geographical barriers, and that the resulting genome conservation over space is potentially applicable to many low-abundance microbial taxa. Our approach shows the value of augmenting amplicon profiling of poorly characterized communities with additional data, such as the physiological characterization of isolates and geochemical analysis, in order to provide context and validation of ecological inferences.

## Supplementary Material

Supplemental material

## References

[1] Hugenholtz P, Pitulle C, Hershberger KL, Pace NR 1998 Novel division level bacterial diversity in a Yellowstone hot spring. J Bacteriol 180:366–376. 9440526 10.1128/jb.180.2.366-376.1998 PMC106892

[2] Portillo MC, Gonzalez JM 2008 Members of the Candidate Division OP10 are spread in a variety of environments. World J Microbiol Biotechnol 25:347–353. doi:10.1007/s11274-008-9895-z.

[3] Dunfield PF, Tamas I, Lee KC, Morgan XC, McDonald IR, Stott MB 2012 Electing a candidate: a speculative history of the bacterial phylum OP10. Environ Microbiol 14:3069–3080. doi:10.1111/j.1462-2920.2012.02742.x. 22497633

[4] Lee KCY, Herbold CW, Dunfield PF, Morgan XC, McDonald IR, Stott MB 2013 Phylogenetic delineation of the novel phylum Armatimonadetes (former candidate division OP10) and definition of two novel candidate divisions. Appl Environ Microbiol 79:2484–2487. doi:10.1128/AEM.03333-12. 23377935 PMC3623213

[5] Lee KC-Y, Dunfield PF, Morgan XC, Crowe MA, Houghton KM, Vyssotski M, Ryan JLJ, Lagutin K, McDonald IR, Stott MB 2011 Chthonomonas calidirosea gen. nov., sp. nov., an aerobic, pigmented, thermophilic micro-organism of a novel bacterial class, Chthonomonadetes classis nov., of the newly described phylum Armatimonadetes originally designated candidate division OP10. Int J Syst Evol Microbiol 61:2482–2490. doi:10.1099/ijs.0.027235-0. 21097641

[6] Tamaki H, Tanaka Y, Matsuzawa H, Muramatsu M, Meng X-Y, Hanada S, Mori K, Kamagata Y 2011 Armatimonas rosea gen. nov., sp. nov., of a novel bacterial phylum, Armatimonadetes phyl. nov, formally called the candidate phylum OP10. Int J Syst Evol Microbiol 61:1442–1447. 20622056 10.1099/ijs.0.025643-0

[7] Im W-T, Hu Z-Y, Kim K-H, Rhee S-K, Meng H, Lee S-T, Quan Z-X 2012 Description of Fimbriimonas ginsengisoli gen. nov., sp. nov. within the Fimbriimonadia class nov., of the phylum Armatimonadetes. Antonie Van Leeuwenhoek 102:307–317. doi:10.1007/s10482-012-9739-6. 22527625

[8] Lee KC, Morgan XC, Dunfield PF, Tamas I, McDonald IR, Stott MB 2014 Genomic analysis of Chthonomonas calidirosea, the first sequenced isolate of the phylum Armatimonadetes. ISME J 8:1522–1533. doi:10.1038/ismej.2013.251. 24477196 PMC4069393

[9] Hu Z-Y, Wang Y-Z, Im W-T, Wang S-Y, Zhao G-P, Zheng H-J, Quan Z-X 2014 The first complete genome sequence of the class Fimbriimonadia in the phylum Armatimonadetes. PLoS One 9:e100794. doi:10.1371/journal.pone.0100794. 24967843 PMC4072686

[10] Stott MB, Crowe MA, Mountain BW, Smirnova AV, Hou S, Alam M, Dunfield PF 2008 Isolation of novel bacteria, including a candidate division, from geothermal soils in New Zealand. Environ Microbiol 10:2030–2041. doi:10.1111/j.1462-2920.2008.01621.x. 18422642

[11] Pruesse E, Quast C, Knittel K, Fuchs BM, Ludwig W, Peplies J, Glöckner FO 2007 SILVA: a comprehensive online resource for quality checked and aligned ribosomal RNA sequence data compatible with ARB. Nucleic Acids Res 35:7188–7196. doi:10.1093/nar/gkm864. 17947321 PMC2175337

[12] Benson DA, Karsch-Mizrachi I, Lipman DJ, Ostell J, Sayers EW 2011 GenBank. Nucleic Acids Res 39:D32–D37. doi:10.1093/nar/gkq1079. 21071399 PMC3013681

[13] Nuñez PA, Romero H, Farber MD, Rocha EPC 2013 Natural selection for operons depends on genome size. Genome Biol Evol 5:2242–2254. doi:10.1093/gbe/evt174. 24201372 PMC3845653

[14] Memon D, Singh AK, Pakrasi HB, Wangikar PP 2013 A global analysis of adaptive evolution of operons in cyanobacteria. Antonie Van Leeuwenhoek 103:331–346. doi:10.1007/s10482-012-9813-0. 22987250

[15] Xie G, Bonner CA, Brettin T, Gottardo R, Keyhani NO, Jensen RA 2003 Lateral gene transfer and ancient paralogy of operons containing redundant copies of tryptophan-pathway genes in Xylella species and in heterocystous cyanobacteria. Genome Biol 4:R14. doi:10.1186/gb-2003-4-2-r14. 12620124 PMC151304

[16] Itoh T, Takemoto K, Mori H, Gojobori T 1999 Evolutionary instability of operon structures disclosed by sequence comparisons of complete microbial genomes. Mol Biol Evol 16:332–346. doi:10.1093/oxfordjournals.molbev.a026114. 10331260

[17] Coleman ML, Sullivan MB, Martiny AC, Steglich C, Barry K, Delong EF, Chisholm SW 2006 Genomic islands and the ecology and evolution of Prochlorococcus. Science 311:1768–1770. doi:10.1126/science.1122050. 16556843

[18] Wang B, Lu L, Lv H, Jiang H, Qu G, Tian C, Ma Y 2014 The transcriptome landscape of Prochlorococcus MED4 and the factors for stabilizing the core genome. BMC Microbiol 14:11. doi:10.1186/1471-2180-14-11. 24438106 PMC3898218

[19] Zheng Y, Szustakowski JD, Fortnow L, Roberts RJ, Kasif S 2002 Computational identification of operons in microbial genomes. Genome Res 12:1221–1230. doi:10.1101/gr.200601. 12176930 PMC186635

[20] Chevreux B, Wetter T, Suhai S 1999 Genome sequence assembly using trace signals and additional sequence information, p 45–56. *In* Computer science and biology. Proceedings of the German Conference on Bioinformatics, GCB '99. GCB, Hannover, Germany.

[21] Darling AE, Mau B, Perna NT 2010 progressiveMauve: multiple genome alignment with gene gain, loss and rearrangement. PLoS One 5:e11147. doi:10.1371/journal.pone.0011147. 20593022 PMC2892488

[22] Markowitz VM, Chen I-MA, Palaniappan K, Chu K, Szeto E, Grechkin Y, Ratner A, Anderson I, Lykidis A, Mavromatis K, Ivanova NN, Kyrpides NC 2010 The integrated microbial genomes system: an expanding comparative analysis resource. Nucleic Acids Res 38:D382–D390. doi:10.1093/nar/gkp887. 19864254 PMC2808961

[23] Langmead B, Salzberg SL 2012 Fast gapped-read alignment with Bowtie 2. Nat Methods 9:357–359. doi:10.1038/nmeth.1923. 22388286 PMC3322381

[24] Quinlan AR 2014 BEDTools: the Swiss-army tool for genome feature analysis. Curr Protoc Bioinformatics 47:11.12.1–11.12.34. 25199790 10.1002/0471250953.bi1112s47 PMC4213956

[25] Edgar RC 2010 Search and clustering orders of magnitude faster than BLAST. Bioinformatics 26:2460–2461. doi:10.1093/bioinformatics/btq461. 20709691

[26] Segata N, Börnigen D, Morgan XC, Huttenhower C 2013 PhyloPhlAn is a new method for improved phylogenetic and taxonomic placement of microbes. Nat Commun 4:2304. 23942190 10.1038/ncomms3304 PMC3760377

[27] Edgar RC 2004 MUSCLE: multiple sequence alignment with high accuracy and high throughput. Nucleic Acids Res 32:1792–1797. doi:10.1093/nar/gkh340. 15034147 PMC390337

[28] Huelsenbeck JP, Ronquist F 2001 MRBAYES: Bayesian inference of phylogenetic trees. Bioinformatics 17:754–755. doi:10.1093/bioinformatics/17.8.754. 11524383

[29] Posada D 2008 jModelTest: phylogenetic model averaging. Mol Biol Evol 25:1253–1256. doi:10.1093/molbev/msn083. 18397919

[30] Mosmann T 1983 Rapid colorimetric assay for cellular growth and survival: application to proliferation and cytotoxicity assays. J Immunol Methods 65:55–63. doi:10.1016/0022-1759(83)90303-4. 6606682

[31] R Development Core Team. 2013 R: a language and environment for statistical computing. R Foundation for Statistical Computing, Vienna, Austria.

[32] American Public Health Association. 2005 Standard methods for the examination of waste & wastewater, centennial edition, 21 Har/Cdr ed. American Public Health Association, Washington, DC.

[33] Edgar RC 2013 UPARSE: highly accurate OTU sequences from microbial amplicon reads. Nat Methods 10:996–998. doi:10.1038/nmeth.2604. 23955772

[34] Caporaso JG, Kuczynski J, Stombaugh J, Bittinger K, Bushman FD, Costello EK, Fierer N, Peña AG, Goodrich JK, Gordon JI, Huttley GA, Kelley ST, Knights D, Koenig JE, Ley RE, Lozupone CA, McDonald D, Muegge BD, Pirrung M, Reeder J, Sevinsky JR, Turnbaugh PJ, Walters WA, Widmann J, Yatsunenko T, Zaneveld J, Knight R 2010 QIIME allows analysis of high-throughput community sequencing data. Nat Methods 7:335–336. doi:10.1038/nmeth.f.303. 20383131 PMC3156573

[35] DeSantis TZ, Hugenholtz P, Larsen N, Rojas M, Brodie EL, Keller K, Huber T, Dalevi D, Hu P, Andersen GL 2006 Greengenes, a chimera-checked 16S rRNA gene database and workbench compatible with ARB. Appl Environ Microbiol 72:5069–5072. doi:10.1128/AEM.03006-05. 16820507 PMC1489311

[36] Caporaso JG, Bittinger K, Bushman FD, DeSantis TZ, Andersen GL, Knight R 2010 PyNAST: a flexible tool for aligning sequences to a template alignment. Bioinformatics 26:266–267. doi:10.1093/bioinformatics/btp636. 19914921 PMC2804299

[37] Price MN, Dehal PS, Arkin AP 2010 FastTree 2—approximately maximum-likelihood trees for large alignments. PLoS One 5:e9490. doi:10.1371/journal.pone.0009490. 20224823 PMC2835736

[38] Steiner A 1977 The Wairakei geothermal area, North Island, New Zealand: its subsurface geology and hydrothermal rock alteration, p 136 *In* New Zealand geological survey bulletin 90. New Zealand Department of Scientific and Industrial Research, Wellington, New Zealand.

[39] Browne PRL 1978 Hydrothermal alteration in active geothermal fields. Annu Rev Earth Planet Sci 6:229–250. doi:10.1146/annurev.ea.06.050178.001305.

[40] Reyes AG 1990 Petrology of Philippine geothermal systems and the application of alteration mineralogy to their assessment. J Volcanol Geotherm Res 43:279–309. doi:10.1016/0377-0273(90)90057-M.

[41] Kanchikerimath M, Singh D 2001 Soil organic matter and biological properties after 26 years of maize-wheat-cowpea cropping as affected by manure and fertilization in a Cambisol in semiarid region of India. Agric Ecosyst Environ 86:155–162. doi:10.1016/S0167-8809(00)00280-2.

[42] Li W, Pan KW, Wu N, Wang JC, Wang YJ, Zhang L 2014 Effect of litter type on soil microbial parameters and dissolved organic carbon in a laboratory microcosm experiment. Plant Soil Environ 60:170–176.

[43] Osman KT 2013 Soils: principles, properties, and management. Springer Netherlands, Dordrecht, the Netherlands.

[44] Reysenbach AL 2001 Class IV. Thermoplasmata class nov., p 335–338. *In* Boone DR, Castenholz RW (ed), Bergey's manual of systematic bacteriology, vol 1: the Archaea and the deeply branching and phototrophic bacteria, 2nd ed Springer-Verlag, New York, NY.

[45] Vetriani C, Jannasch HW, MacGregor BJ, Stahl DA, Reysenbach AL 1999 Population structure and phylogenetic characterization of marine benthic Archaea in deep-sea sediments. Appl Environ Microbiol 65:4375–4384. 10508063 10.1128/aem.65.10.4375-4384.1999 PMC91581

[46] Stieglmeier M, Klingl A, Alves RJE, Rittmann SK-MR, Melcher M, Leisch N, Schleper C 2014 Nitrososphaera viennensis gen. nov., sp. nov., an aerobic and mesophilic, ammonia-oxidizing archaeon from soil and a member of the archaeal phylum Thaumarchaeota. Int J Syst Evol Microbiol 64:2738–2752. doi:10.1099/ijs.0.063172-0. 24907263 PMC4129164

[47] Yakimov MM, La Cono V, Slepak VZ, La Spada G, Arcadi E, Messina E, Borghini M, Monticelli LS, Rojo D, Barbas C, Golyshina OV, Ferrer M, Golyshin PN, Giuliano L 2013 Microbial life in the Lake Medee, the largest deep-sea salt-saturated formation. Sci Rep 3:3554. 24352146 10.1038/srep03554 PMC3867751

[48] Kato S, Ohkuma M, Yamagishi A 2015 Intra-field variation of prokaryotic communities on and below the seafloor in the back-arc hydrothermal system of the southern Mariana trough, p 301–311. *In* Ishibashi J, Okino K, Sunamura M (ed), Subseafloor biosphere linked to hydrothermal systems. Springer Japan, Tokyo, Japan.

[49] Kan J, Clingenpeel S, Macur RE, Inskeep WP, Lovalvo D, Varley J, Gorby Y, McDermott TR, Nealson K 2011 Archaea in Yellowstone Lake. ISME J 5:1784–1795. doi:10.1038/ismej.2011.56. 21544103 PMC3197168

[50] Norris PR, Clark DA, Owen JP, Waterhouse S 1996 Characteristics of Sulfobacillus acidophilus sp. nov. and other moderately thermophilic mineral-sulphide-oxidizing bacteria. Microbiology 142:775–783. doi:10.1099/00221287-142-4-775. 8936305

[51] Cleaver AA, Burton NP, Norris PR 2007 A novel Acidimicrobium species in continuous cultures of moderately thermophilic, mineral-sulfide-oxidizing acidophiles. Appl Environ Microbiol 73:4294–4299. doi:10.1128/AEM.02658-06. 17468267 PMC1932778

[52] Dunfield PF, Yuryev A, Senin P, Smirnova AV, Stott MB, Hou S, Ly B, Saw JH, Zhou Z, Ren Y, Wang J, Mountain BW, Crowe MA, Weatherby TM, Bodelier PLE, Liesack W, Feng L, Wang L, Alam M 2007 Methane oxidation by an extremely acidophilic bacterium of the phylum Verrucomicrobia. Nature 450:879–882. doi:10.1038/nature06411. 18004300

[53] Bankevich A, Nurk S, Antipov D, Gurevich AA, Dvorkin M, Kulikov AS, Lesin VM, Nikolenko SI, Pham S, Prjibelski AD, Pyshkin AV, Sirotkin AV, Vyahhi N, Tesler G, Alekseyev MA, Pevzner PA 2012 SPAdes: a new genome assembly algorithm and its applications to single-cell sequencing. J Comput Biol 19:455–477. doi:10.1089/cmb.2012.0021. 22506599 PMC3342519

[54] Guo L, Brügger K, Liu C, Shah SA, Zheng H, Zhu Y, Wang S, Lillestøl RK, Chen L, Frank J, Prangishvili D, Paulin L, She Q, Huang L, Garrett RA 2011 Genome analyses of Icelandic strains of Sulfolobus islandicus, model organisms for genetic and virus-host interaction studies. J Bacteriol 193:1672–1680. doi:10.1128/JB.01487-10. 21278296 PMC3067641

[55] Cava F, Hidalgo A, Berenguer J 2009 Thermus thermophilus as biological model. Extremophiles 13:213–231. doi:10.1007/s00792-009-0226-6. 19156357

[56] Huber H, Prangishvili D 2006 Sulfolobales, p 23–51. *In* Dworkin M, Falkow S, Rosenberg E, Schleifer K-H, Stackebrandt E (ed), The prokaryotes: vol 3: Archaea. Bacteria: Firmicutes, Actinomycetes. Springer New York, New York, NY.

[57] Villanueva L, Sinninghe Damsté JS, Schouten S 2014 A re-evaluation of the archaeal membrane lipid biosynthetic pathway. Nat Rev Microbiol 12:438–448. doi:10.1038/nrmicro3260. 24801941

[58] Berg IA, Kockelkorn D, Ramos-Vera WH, Say RF, Zarzycki J, Hügler M, Alber BE, Fuchs G 2010 Autotrophic carbon fixation in archaea. Nat Rev Microbiol 8:447–460. doi:10.1038/nrmicro2365. 20453874

[59] Könneke M, Schubert DM, Brown PC, Hügler M, Standfest S, Schwander T, Schada von Borzyskowski L, Erb TJ, Stahl DA, Berg IA 2014 Ammonia-oxidizing archaea use the most energy-efficient aerobic pathway for CO_2_ fixation. Proc Natl Acad Sci U S A 111:8239–8244. doi:10.1073/pnas.1402028111. 24843170 PMC4050595

[60] Cuecas A, Portillo MC, Kanoksilapatham W, Gonzalez JM 2014 Bacterial distribution along a 50°C temperature gradient reveals a parceled out hot spring environment. Microb Ecol 68:729–739. doi:10.1007/s00248-014-0437-y. 24889287

[61] Stern A, Sorek R 2011 The phage-host arms race: shaping the evolution of microbes. Bioessays 33:43–51. doi:10.1002/bies.201000071. 20979102 PMC3274958

[62] Kobayashi I 2001 Behavior of restriction-modification systems as selfish mobile elements and their impact on genome evolution. Nucleic Acids Res 29:3742–3756. doi:10.1093/nar/29.18.3742. 11557807 PMC55917

[63] Herbold CW, Lee CK, McDonald IR, Cary SC 2014 Evidence of global-scale aeolian dispersal and endemism in isolated geothermal microbial communities of Antarctica. Nat Commun 5:3875. 24846491 10.1038/ncomms4875

[64] Dyall-Smith ML, Pfeiffer F, Klee K, Palm P, Gross K, Schuster SC, Rampp M, Oesterhelt D 2011 Haloquadratum walsbyi: limited diversity in a global pond. PLoS One 6:e20968. doi:10.1371/journal.pone.0020968. 21701686 PMC3119063

[65] Grote J, Thrash JC, Huggett MJ, Landry ZC, Carini P, Giovannoni SJ, Rappé MS 2012 Streamlining and core genome conservation among highly divergent members of the SAR11 clade. mBio 3(5):. doi:10.1128/mBio.00252-12. PMC3448164 22991429

