# Supplementary material for: The Chthonomonas calidirosea Genome Is Highly Conserved across Geographic Locations and Distinct Chemical and Microbial Environments in New Zealand's Taupō Volcanic Zone
Source: Appl Environ Microbiol. 2016 May 31;82(12):3572–81. doi: 10.1128/AEM.00139-16 (PMC4959169; doi:10.1128/AEM.00139-16)
Supplement: Supplemental material [file supp_82_12_3572__index.html]

Supplemental material 

# The Chthonomonas calidirosea Genome Is Highly Conserved across Geographic Locations and Distinct Chemical and Microbial Environments in New Zealand's Taupō Volcanic Zone

## Supplemental material

- Supplemental file 1 -

  Supplemental text (*Chthonomonas* phylotypes found outside of the Taupō Volcanic Zone [TVZ]; calculation of pairwise nucleotide distances of functionally related genes within genomes; assembly quality control; identification of putative genes in unmapped reads; identification of gene homologs across *C. calidirosea* isolates [code; conservation of genomic content]; comparison of genetic divergence in *S. islandicus* and *T. thermophiles*; community 16S rRNA gene-targeted sequencing and processing); Fig. S1: positional dispersal of genes involved in four biosynthetic pathways within representative bacterial genomes; Fig. S2: synteny of *C. calidirosea* genome assemblies; Fig. S3: quality assessments of paired-end genome assemblies; Fig. S4: histograms of coverage of genes for each isolate; Table S11: distribution of within-own-genome read coverage of called genes, in fractions; Table S12: fraction of high-coverage genes in each isolate that are not shared with other isolates; Fig. S5: progressiveMauve genome alignment of *Chthonomonas calidirosea* isolates showing a high degree of synteny among the genomes (A), of three *Thermus thermophilus* isolates (B), and of two *Sulfolobus islandicus* isolates from Yellowstone National Park showing regions of genome rearrangement and areas of low sequence similarity within the locally collinear blocks (C); Fig. S6: correlation of BIOLOG phenotype microarray response between *C. calidirosea* isolates; Fig. S7: mineral composition of sample sites; Fig. S8: observed OTUs in the four sample sites; Fig. S9: map of the TVZ; Fig. S10: alignments of putative HGT regions.

  PDF, 3.0M
- Supplemental file 2 -

  Table S1: list of conserved genes used for phylogenetic inference; Table S2: relative weight percentage of major metal oxides detected in the samples from the four sites using XRF; Table S3: OTUs; Table S4: relative taxa abundance of the four communities containing *C. calidirosea*; Table S5: beta diversity of the four communities, measured using Bray-Curtis, weighted and unweighted Unifrac matrices; Fig. S6: coverage of homologs from mapping reads onto protein-coding genes; Fig. S7: assessment of total bias from using the T49T reference genome as the assembly backbone; Fig. S8: total number and average base differences of 308 conserved genes of *Thermus thermophilus* isolates JL-18, HB-27, and BH-8 and ATCC 33923 and of 147 conserved genes of *Sulfolobus islandicus* isolates Y.N.15.51 Y.G.57.14, and nucleotide divergence of 16S rRNA genes of those strains; Fig. S9: colorimetric redox responses of *C. calidirosea* isolates with BIOLOG PM1 phenotype microarray; Fig. S10: pairwise discontiguous megaBLAST comparison of 16S rRNA gene sequences between *Armatimonadetes* OTUs identifies in a Thailand hot spring and *Armatimonadetes* classes (*Armatimonas rosea* YO-36T, *Fimbriimonas ginsengisoli* Gsoil 348T, and *Chthonomonas calidirosea* T49T).

  XLSX, 811K
