# Supplementary material for: The Chthonomonas calidirosea Genome Is Highly Conserved across Geographic Locations and Distinct Chemical and Microbial Environments in New Zealand's Taupō Volcanic Zone
Source: Appl Environ Microbiol. 2016 May 31;82(12):3572–81. doi: 10.1128/AEM.00139-16 (PMC4959169; doi:10.1128/AEM.00139-16)
Supplement: Supplemental material [file AEM.00139-16_zam999117198so1.pdf]

# Supplementary Materials

## 1 *Chthonomonas* spp. phylotypes found outside of the Taupō Volcanic Zone (TVZ)

The 16S rRNA gene sequence of *C. calidirosea* T49$^T$ was searched against the NCBI non-redundant (nr) nucleotide database (1) (accessed December 2015) and the Earth Microbiome Project (2) dataset (January 2014 release) in order to identify closely-related phylotypes.

Nucleotide BLAST searches of the NCBI nr database (excluding *C. calidirosea* phylotypes previously identified in the TVZ) identified several sequences (KM102598.1, KM102600.1, KM102602.1, KM102610.1, KM102611.1, KM102614.1, KM102615.1, and KM102618.1) from volcanic soil in Parícutin, Mexico as being closely-related (~ 93% sequence identity based on ClustalW pairwise analyses of the available 900 bp sequences) to strain T49$^T$. However, these sequences are shorter (~60% coverage of the full gene sequence) than other BLAST hits, and therefore yielded lower bit scores. The closest hit from the NCBI nr database with the highest bit score (excluding *C. calidirosea* sequences from the TVZ) was "Uncultured bacterium clone T2-75" (accession number GQ441872) from marine microbial mats, which exhibited 91 % 16S rRNA gene sequence identity with *C. calidirosea* T49$^T$. Nucleotide BLAST searches of the EMP NGS dataset did not identify closely-related phylotypes outside of the TVZ. Furthermore, none of the closest hits were associated with thermophilic environments. The closest hit from the EMP dataset was "New.61.CleanUp.ReferenceOTU37399 KA1F.E.13_00114", with 88 % V4 region sequence identity. This OTU originated from a temperate soil metagenomics project (http://metagenomics.anl.gov/linkin.cgi?project=2560).

## 2 Calculation of pairwise nucleotide distances of functionally-related genes within genomes

To quantitatively compare the degree of dispersal of functionally-related genes traditionally structured within operons (i.e. "genome disorganization") in the genome of *C. calidirosea* T49$^T$ to a variety other bacterial species, we calculated the pairwise Euclidean nucleotide distances between the genes involved in four common metabolic pathways in a range of genomes both closely and distantly related to *C. calidirosea* (**Figure S1)**. We selected the biosynthetic pathways for tryptophan (trpABCDE), histidine (hisGBIEDAFH), purine (purEBCLFMNHD), and leucine (leuABCD) because they are extremely well-characterized within model organisms such as *E. coli* and *B. subtilis* (in which they are organized in operons) (3–7), and are near-ubiquitous in bacteria. We selected both genomes within phyla closely-related to *C. calidirosea* (e.g. *Chloroflexi, Deinococcus-Thermus, Actinobacteria, and Cyanobacteria*) and more distantly-related taxa (e. g. *Proteobacteria* and *Thermotogae*).

For this comparison, only high-quality closed genomes containing a single chromosome were selected, to avoid comparison of genetic distances between two different chromosomes. To determine putative orthologs of the targeted genes, the target genes from each pathway were identified within *E. coli* strain K12, and these were used to identify homologues within the other genomes using reciprocal blastp searches within the IMG system (8). Coordinates and genome sizes were downloaded from IMG. For each taxa and each gene pathway, all possible pairs of genes were generated, and the shortest pairwise Euclidean nucleotide distance was calculated between each gene pair in the pathway. Specifically, for each gene pair, the shorter of two paths between the 5' ends of two genes on the chromosome was calculated, and these were summed for each pathway and each genome. A single genome coordinate system was used, and strand orientation of genes was ignored. Pairwise distances were calculated using a custom Python script (operon.py, available at http://bitbucket.org/biobakery/chthonomonas ) and plotted via ggplot2 (9) in R (10) on a log10 scale. The phylogenies of the bacterial phyla outlined by the cladogram (**Figure S1**) were derived from the iTOL project (11) and Lee *et al*. (12).

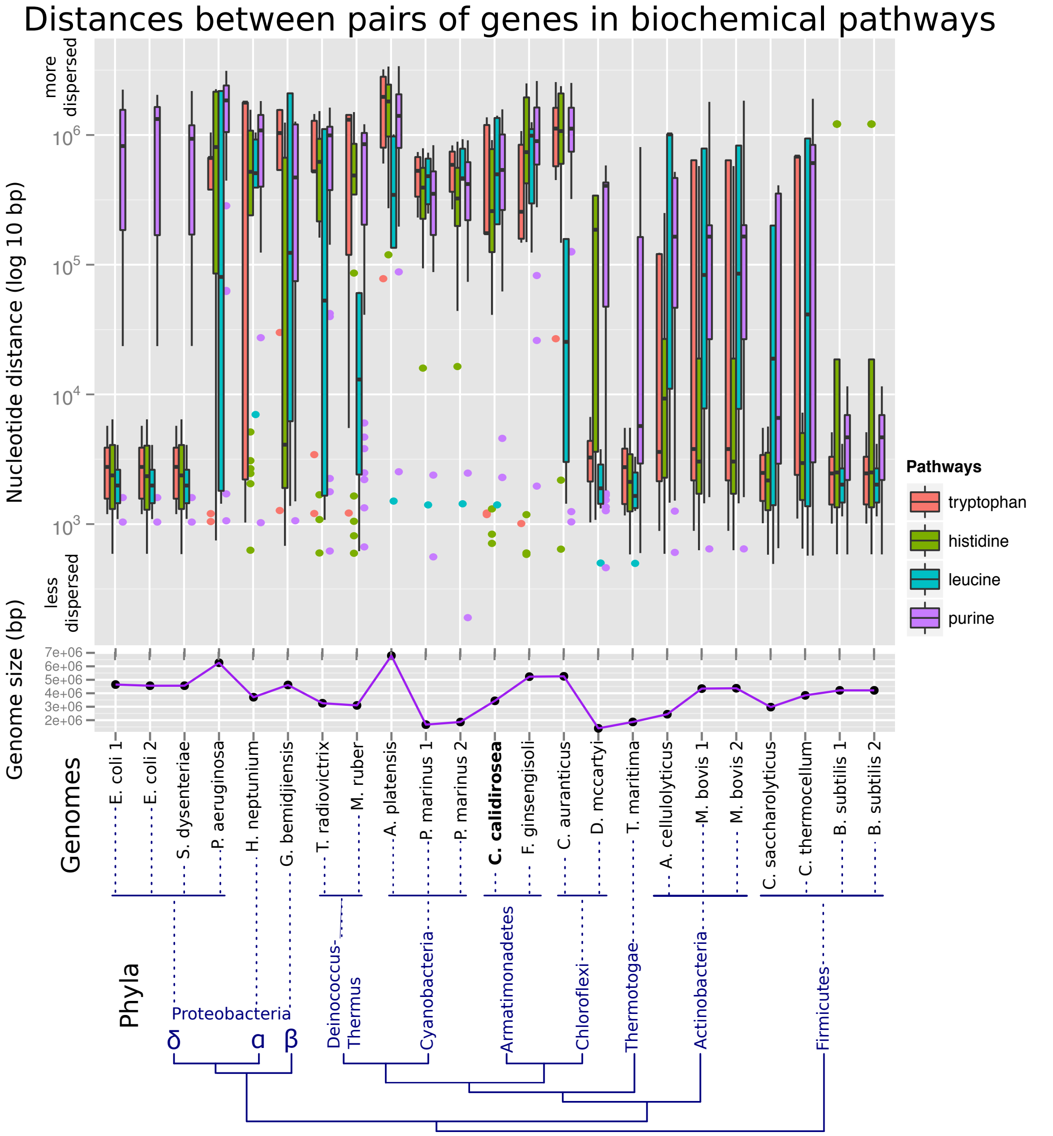


**Figure S1 - The positional dispersal of genes involved in four biosynthetic pathways within representative bacterial genomes.** (Top) For each representative genome, for each of four biosynthetic pathways, we calculated the shortest Euclidean distance between each pair of genes in the pathway. The Tukey boxplots show the distributions of pairwise distances between the genes in the pathways. The upper and lower whiskers of the boxplots indicate 1.5 interquartile ranges (IQR), and outliers beyond 1.5 IQR are plotted as points. (Center) The length (bp) of each representative genome. (Bottom) The phylogenetic relationships of the representative genomes. The representative genomes are (right to left): *Escherichia coli* K-12 W3110 (13), *Escherichia coli* BL21(DE3) (14), *Shigella dysenteriae* sv. 1 Sd197 (15), *Pseudomonas aeruginosa* PAO1 (16), *Hyphomonas neptunium* ATCC 15444$^T$ (17), *Geobacter bemidjiensis* Bem$^T$ DSM 16622$^T$ (18), *Truepera radiovictrix* RQ-24$^T$ DSM 17093$^T$ (19), *Meiothermus ruber* 21$^T$ DSM 1279$^T$ (20), *Arthrospira platensis* NIES-39 (21), *Prochlorococcus marinus* AS9601 (22), *Prochlorococcus marinus* NATL1A (22), *Chthonomonas calidirosea* T49$^T$ DSM 23976$^T$ (12), *Fimbriimonas ginsengisoli* Gsoil 348$^T$ (23), *Chloroflexus aurantiacus* J-10-fl$^T$ (24), *Dehalococcoides mccartyi* CBDB1 (25), *Thermotoga maritima* MSB8$^T$ (26), *Acidothermus cellulolyticus* 11B$^T$ (27), *Mycobacterium bovis* AF2122/97 (28), *Mycobacterium bovis* BCG Tokyo 172 (29), *Caldicellulosiruptor saccharolyticus* DSM 8903$^T$ (30), *Clostridium thermocellum* ATCC 27405$^T$ (31), *Bacillus subtilis* subtilis 168$^T$ (32), and *Bacillus subtilis* subtilis 6051-HGW (33).

## 2.1 Phylum-dependent dispersal of genes within prokaryotic genomes (Figure S1). Additional context leading to this research.

The clustering of functionally-related genes (gene clusters), often as cotranscribed units (operons), is frequently observed and is thought to be typical for gene organization and regulation in prokaryotes (34). However, functionally-related or “functionally-linked” genes may occur in close proximity despite a lack of classic operon cotranscription structure (35, 36). The underlying mechanisms driving operon formation remained poorly understood. The propensity of functionally-related genes to cluster in proximity within a genome has been utilized to infer function of putative genes (37, 38). However, many regions within the genome of *C. calidirosea* T49$^T$ were not well-organized into gene clusters and operons (12).

Bacterial species exhibit different degrees of operonic organization. To compare the extent of dispersal of functionally-related genes (i.e. apparent genomic disorganization) of T49 to that of other prokaryotic species, we calculated the Euclidean nucleotide distances between pairs of genes in several common metabolic pathways (trp, his, leu, and pur), and compared the distribution of distances in several different organisms (**Figure S1**). The distributions of distances were used as an indicator of pathway dispersal. Model organisms such as *E. coli* and *B. subtilis*, in which these pathways were first described, showed high degrees of organization (i.e. small Euclidean distances), as most of the genes in each pathway were tightly organized within a single operon. Similar tight organization is found in species in other lineages, such as the trp genes in *Dehalococcoides mccartyi*. However, most genomes in our dataset exhibited a wider organizational distribution, indicating partial clustering of genes. Examples of this group of genes include the purine biosynthetic genes for *Clostridium thermocellum* (nine pur genes, of which six (purDHMNFE) are in a cluster).

Finally, some genomes exhibited overall large distance between these genes. For example, the set of purine biosynthesis genes in *C. calidirosea* comprises eight genes, only two of which (purLM) are adjacent. Based on the dataset of genomes and pathways analyzed, functionally-related genes within the genomes of both the *Cyanobacteria* and *Armatimonadetes* appear particularly dispersed relative to other taxa.

This apparent disorganization of cyanobacteria is supported by previous genomic analysis of multiple samples of *Prochlorococcus marinus* MED4 (39). The transcriptomic analysis has shown that the majority (63.0 %) of the operons identified consisted of only two genes, with an average operon length (AOL, in number of genes within the operon) of 2.7. Comparatively, this is among the shortest average operon length analyzed by Zheng et al., (40), which identified *E. coli* and *B. subtilis* (AOL 3.0 and 3.1) as among the most highly organized, while *Borrelia burgdorferi* and the cyanobacteria *Synechocystis* (both with AOL of 2.5) were among the least organized genomes. Frequent gene translocation or poor enzyme annotation were speculated to be the causes of operon dispersal. This extensive gene disassociation included the tryptophan operon commonly found in many bacterial species (41). The study on *Prochlorococcus* MED4 also concluded that the more conserved genes among the genomes tended to be within operons (39), mirroring conservation of functionally-related genomic neighborhoods (i.e. genes closer to each other tended to remain closer to each other between genomes) found in other studies (42, 43). Since non-core genes were less likely to be within operons (39, 44), we suspected the many *C. calidirosea* genes which lacked apparent functionally-related neighbors represent the flexible segment of its genome. In order to investigate the nature of core vs. non-flexible genes among the apparent disorganized genome, we conducted a comparative genomic analysis on multiple samples of *C. calidirosea* isolated from multiple geothermal sites within the TVZ.

## 3 Assembly quality control

We assessed the quality and synteny of the paired-end sequenced P488 and WRG1.2 genome assemblies by mapping paired-end reads onto the respective assembled genome sequences with Hagfish (https://github.com/mfiers/hagfish/). This process was not possible for the TKA4.10 genome, as the Ion Torrent sequencing used a single-ended sequencing protocol. The vast majority of read pairs for both genomes mapped within the insertion size range (300-1000 bp, assessed via Bioanalyzer) of the Illumina libraries (**Figure S3**), indicating shared synteny over most genomic regions between P488, WRG1.2, and the reference T49$^T$ genome. Nevertheless, the mapped genome coverage plot also showed a small number of regions with poor read coverage and regions with mapped reads longer than expected pair-end length, indicating possible hypervariable regions, genomic rearrangement breakpoints, or assembly problems such as repetitive regions or mismatches between reference and the genome. We also assessed contigs that were not assembled into the scaffold (see section 4). Additionally, in order to assess potential biases from using MIRA assembler and the reference genome, the consensus sequence of the mapped assemblies were compared using CONTIGuator2 (45) with contigs *de novo* assembled by SPAdes assembler (**Figure S2**). The resulting assemblies were highly similar to those produced by MIRA. The contigs showed no indication of genome rearrangement breakpoints and few putative insertions/deletions (indels). Compared to the reference genome, most of the putative indels were located at the edge of the contigs, suggesting assembly artifacts due to gaps in the reference genome or repetitive sequences. The three large putative insertions found only in P488 contained genes found in T49$^T$ (e.g. hydroxypyruvate isomerase and prepilin protein), which may suggest assembly error or potential gene duplication sites. Overall, the *de novo* and reference-based assemblies were highly comparable, indicating high overall genome synteny and low isolate-variant gene contents.

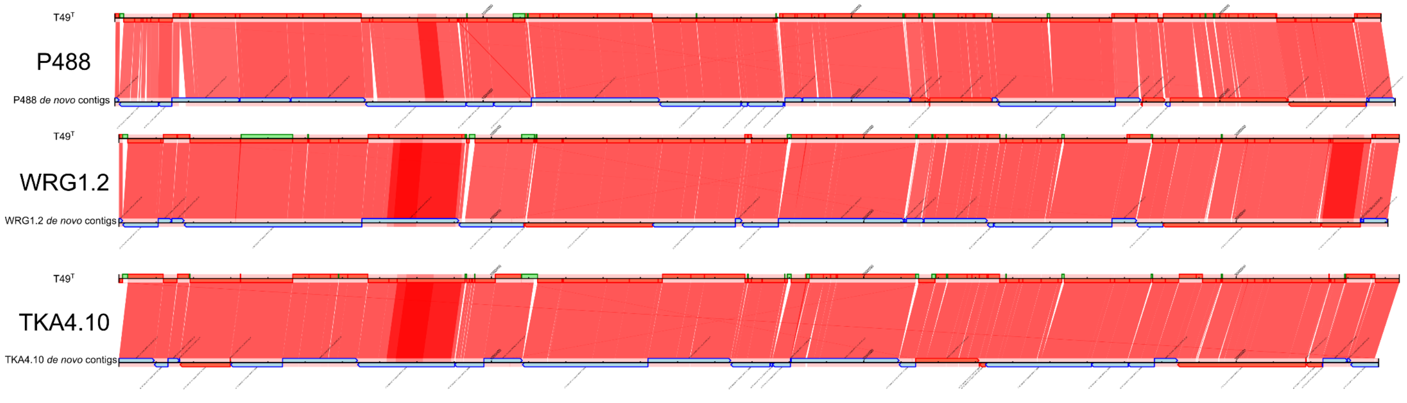


**Figure S2 - Synteny of *C. calidirosea* genome assemblies**. P488, WRG1.2, TKA4.10 *de novo* assembled contigs were mapped against the T49$^T$ genome consensus sequence. The contigs of the three isolates showed high degrees of synteny, with no indication of genome rearrangement breakpoints. Most of the putative insertions/deletions were located at the edge of the contigs, suggesting assembly artifacts due to gaps in the reference genome or repetitive sequences. The three large putative insertions found only in P488 contained genes found in T49$^T$ (e.g. hydroxypyruvate isomerase and prepilin protein), which may indicate assembly error or potential gene duplication sites.

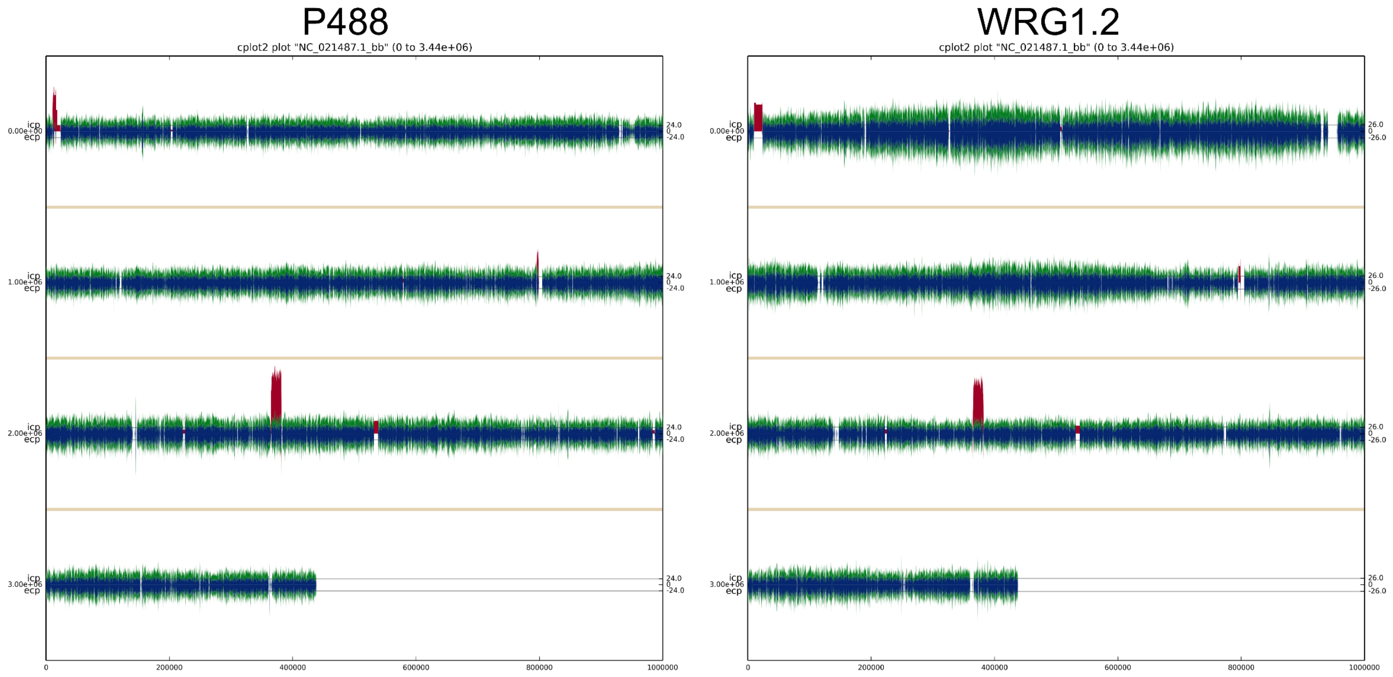


**Figure S3 - Quality assessments of paired-end genome assemblies.** Genome coverage plot generated with Hagfish (https://github.com/mfiers/hagfish). Inclusive coverage and exclusive coverage histogram plots (above and below the center line respectively) indicate the number of P488 or WRG1.2 paired-end reads mapped onto the reference T49$^T$ genome. The inclusive coverage histogram includes the sequence between two paired-end reads, while the exclusive coverage histogram only covers the area of the actual pair-end reads. Green bars indicate read-pairs distance within expected range, blue bars indicate read-pairs shorter than expected paired-end distance, and red bars indicate read-pairs longer than expected distance. Regions with no pair-end data are shown without any peaks. Paired end violation of insertion size may be misassembled due to repetitive regions, regions of sequence not found in the reference genome, or a rearrangement breakpoint. Overall, the plots showed read pairs within expected insertion size range (green peaks). TKA assembly was not analyzed because the genomes was sequenced with the Ion Torrent platform and therefore not pair-ended.

# 4 Identification of putative genes in unmapped reads

Reads that were not successfully assembled by MIRA with strain T49$^T$ used as the reference genome underwent a second round of *de novo*, per-genome assembly with MIRA. All resulting contigs from this second round of assembly that were ≥ 500 bp in length and had more than 1/3 of average coverage of the *de novo* assemblies were searched against the NCBI nr database using default blastn parameters in order to locate putative genes that were unassembled and not present in T49$^T$ (**Table S7**). These selection criteria were chosen because very short nucleotide sequences are more likely to represent repetitive motifs than actual genes.

# 5 Identification of gene homologs across *C. calidirosea* isolates

## 5.1 Code

All code and source files for this project are available at http://bitbucket.org/biobakery/chthomononas.

## 5.2 Conservation of genomic content

We used UCLUST (usearch7.0.1090_i86osx32 -cluster_fast all.fna -id 0.95 -centroids nr2.fasta --log usearch.log --uc results2.uc) to cluster the amino acid sequences of all annotated genes at 95 % similarity. This assessed whether the genome of each isolate was annotated to contain highly-similar genes. There were 3029 unique gene clusters; 2719 (89.7 %) of these genes were present in all four isolates; 141 clusters (4.7 % of genes) were isolate-specific (**Table S7**).

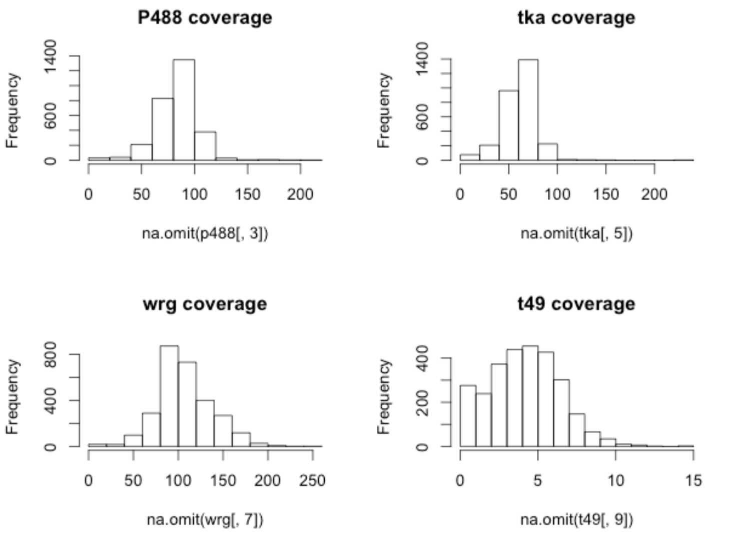


**Figure S4 - Histograms of coverage of genes for each isolate.** Strain T49$^{T}$, which is 454 sequenced, is not covered to sufficient depth for IQR-based filtering.

Because genomes could contain genes that were not properly annotated (the T49$^{T}$ genome in particular contained some gaps, and was assembled and annotated prior to the other isolates), we applied a second method to assess gene conservation between isolates. We assessed the read coverage of each gene in each isolate. In a pairwise manner, we used Bowtie2 (46) to align the sequencing reads from each isolate to each isolate's set of protein-coding genes. We then calculated the median coverage across all bases for each gene using BEDtools (47). Low-coverage outlier genes were defined as those genes with: coverage < (25$^{th}$ percentile - (2 × interquartile range)) in isolates TKA, P488, and WRG (**Figure S4**). In all isolates but T49$^{T}$, 98-99.4 % of the annotated genes in each genome were well-covered. The remaining 0.5 to 2 % of poorly-covered genes are potential candidates for misannotation (**Table S11**).

In strain T49$^{T}$, which was sequenced by 454 sequencing to an average depth of 7x, 7 % of genes had no mapped reads or median coverage 0. It was therefore not possible to apply the IQR-based coverage filter applied to the other isolates. For the purposes of further calculation, we did not filter any T49$^{T}$ genes within strain T49$^{T}$ (all original annotated protein-coding genes are assumed to be present), but only calculated whether protein-coding genes in other isolates had T49$^{T}$ read coverage for comparison. Genes from other isolates were counted as 'present' within the T49$^{T}$ genome if their median coverage in T49$^{T}$ was >= 1. While this parameter is reasonably sensitive, it will result in much higher type II error than the comparisons for the other isolates due to its basis on much shallower sequencing coverage. **Table S6** summarizes coverage of genes between isolate strains, as well as the homolog groups to which each gene belongs. Overall, genomic content was highly conserved between isolates (**Table S12**). Isolates had from 2823 to 2875 protein-coding genes, which corresponded to 3029 unique gene clusters. There was a core pangenome of ~2600 genes with consistent coverage across all genes. With the exception of T49$^{T}$ (for which, as previously discussed, shared genes were underestimated due to shallow sequencing), only ~0.5 % to 1.5 % of genes were not in common between any two isolates. There were a total of 51 apparently isolate-unique genes among the four isolates (totalisolatePresent = 1); the majority (32/51) of these were hypothetical proteins. Of the remainder, the most common functions were related to DNA modification (e.g. restriction enzymes, methylation).

**Table S11 - Distribution of within-own-genome read coverage of called genes, in fractions. MC = Median coverage.**

| Isolates | Coverage | | | |
|---|---|---|---|---|
| | # original protein-coding genes | MC = 0 | MC => 1 | MC >= 25th percentile - 2 IQR |
| T49 | 2878 | 0.068 | 0.932 | 100.000 |
| WRG | 2856 | 0.002 | 0.998 | 0.994 |
| P488 | 2877 | 0.001 | 0.999 | 0.983 |
| TKA | 2886 | 0.014 | 0.986 | 0.979 |

**Table S12 - Fraction of high-coverage (Hicov) genes in each isolate that are not shared with each other isolates**

| Isolates | Not in P488 | Not in TKA | Not in WRG | Not in T49 | Total Hicov genes |
|---|---|---|---|---|---|
| T49 | 0.024 | 0.023 | 0.019 | 0.000 | 2877 |
| WRG | 0.016 | 0.011 | 0.000 | 0.076 | 2840 |
| P488 | 0.000 | 0.013 | 0.009 | 0.071 | 2828 |
| TKA | 0.010 | 0.000 | 0.003 | 0.063 | 2824 |

Furthermore, the assessment of isolate-variant genes were not biased by unassembled reads due to disagreement with the reference genome. Genes in contigs *de novo* assembled from unmapped reads (see above section 3) showed only few DNA-related genes such as restriction enzymes (**Table S7**).

## *6* Comparison of genetic divergence in *S. islandicus* and *T. thermophilus*

We compared the genomic divergence of *C. calidirosea* to that of two other well-studied thermophiles, *Thermus thermophilus* and *Sulfolobus islandicus*, which with the exception to *T. thermophilus* strain JL-18, were isolated across geographic scales comparable to those of *C. calidirosea* in this study. The two *S. islandicus* isolates were isolated from sites approximately 4.7 km apart in Yellowstone National Park (48). This distance was inferred from the site description between Norris Geyser Basin (Y.N.15.51, GPS: 44.728302, -110.704405) and Geyser Creek (Y.G.57.14, GPS: 44.699641, -110.74572). The three Japanese *T. thermophilus* isolates were isolated over a distance of approximately 17 km, from the Izu peninsula in Japan (based on (49–51), and ATCC record therein). The distance was inferred from the name of the sites where the strains were first isolated, namely Mine hot spring (HB-8 and HB-27, GPS: 34.757322, 138.982381. HB-8 isolation site was known and HB-27 was one of the strains isolated by Oshima and Imahori from the same location (52)) and Atagawa Hot Spring (ATCC 33923 GPS: 34.819446, 139.069788). Strain JL-18 was isolated from Nevada, USA (53). Strain alignments with ProgressiveMauve are shown in (**Figure S5**).

A

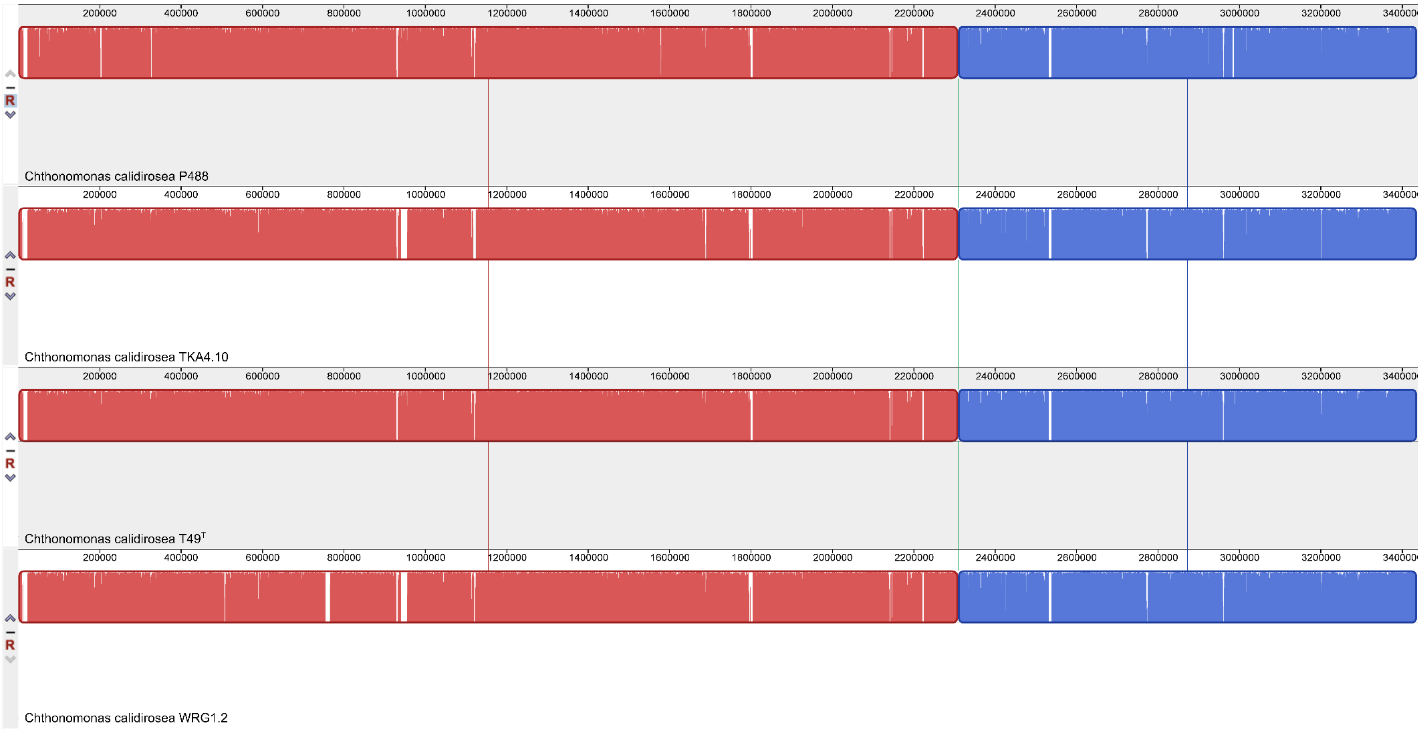
Chthonomonas calidirosea P488
Chthonomonas calidirosea TKA4.10
Chthonomonas calidirosea T49T
Chthonomonas calidirosea WRG1.2

B

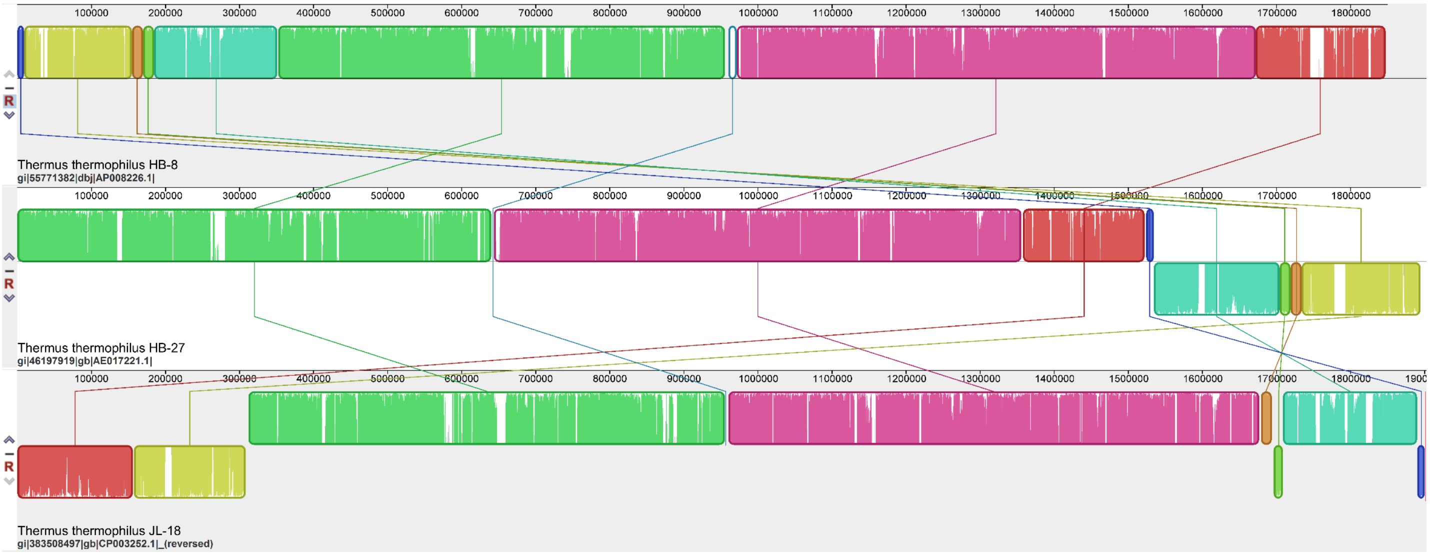
Thermus thermophilus HB-8
gi|55771382|dbj|AP008226.1|
Thermus thermophilus HB-27
gi|46197919|gb|AE017221.1|
Thermus thermophilus JL-18
gi|383508497|gb|CP003252.1|_(reversed)

C

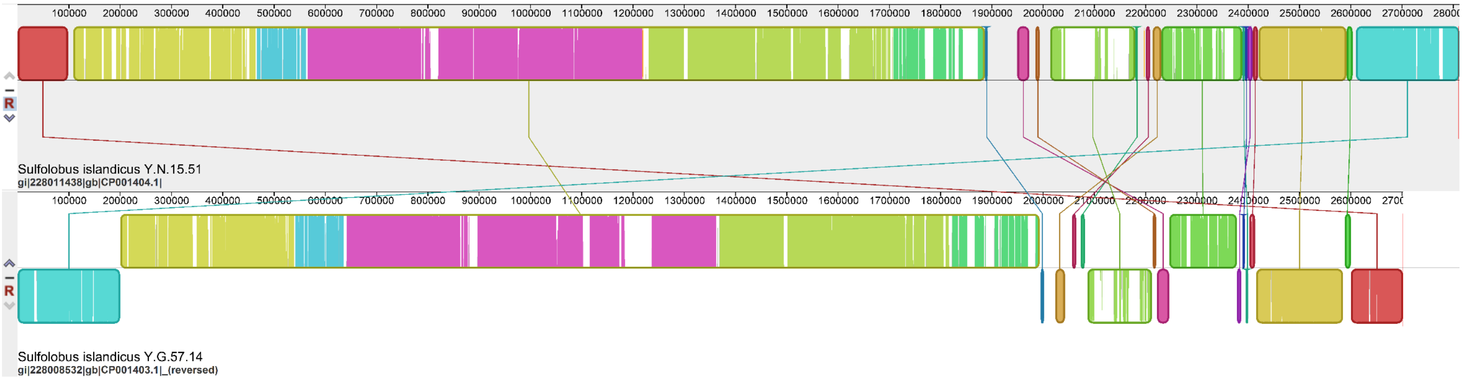

Sulfolobus islandicus Y.N.15.51
gi|228011438|gb|CP001404.1|
Sulfolobus islandicus Y.G.57.14
gi|228008532|gb|CP001403.1|_(reversed)

**Figure S5. (A)** – progressiveMauve (54) genome alignment of *Chthonomonas calidirosea* isolates showing a high degree of synteny among the genomes. The green line shows the location of a putative HGT region examined in Supplementary Figure S6 (CCALI_00803 – CCALI_00807). Similarity plot within each Locally Collinear Block (LCB) indicates the sequence similarity among the genomes. The genomic sequences were assembled with MIRA (55) using T49$^T$ genome as reference. **B) –** progressiveMauve (54) genome alignment of three *Thermus thermophilus* isolates. Isolate HB-8 and HB-27 were isolated from the Izu Peninsula, Japan (49, 50), while isolate JL-18 was isolated from Nevada, USA (53). The alignment shows the sites of rearrangements among the genomes. Note the position of the red Locally Collinear Block (LCB) in relation to other LCBs. The position of the LCBs above or below the median line for each genome indicates the relative sequence direction compared to the reference genome (HB-8). Similarity plot within each LCB indicates the sequence similarity among the genomes. **(C)** – progressiveMauve (54) genome alignment of two *Sulfolobus islandicus* isolates from Yellowstone National Park, USA (48) showing regions of genome rearrangement and areas of low sequence similarity within the Locally Collinear Blocks (LCBs).

# 7 BIOLOG Phenotypic Microarray

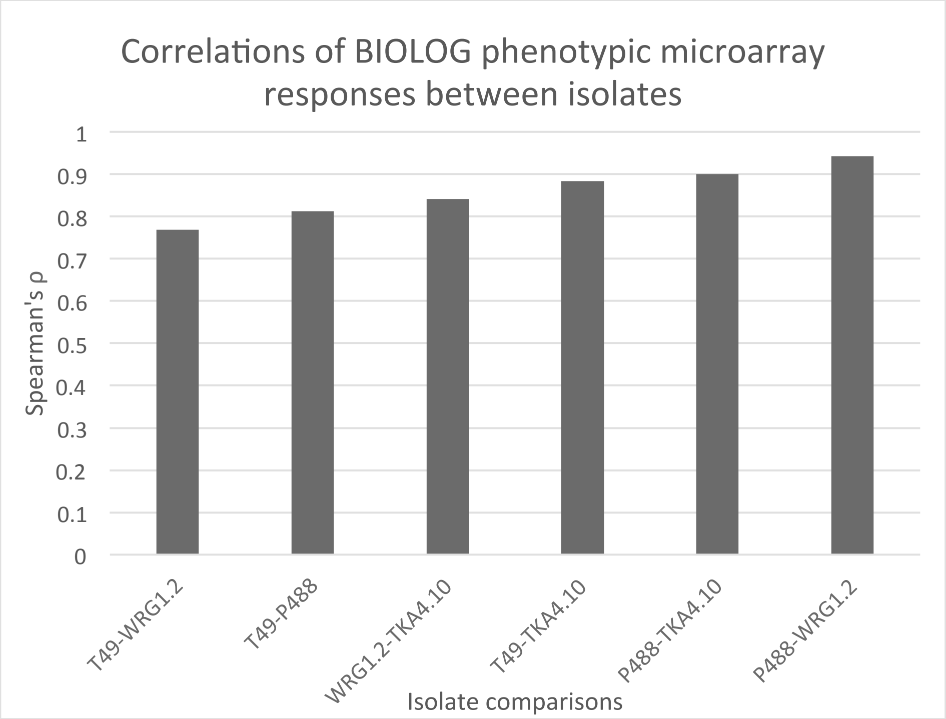


**Figure S6.– Correlation of BIOLOG phenotype microarray response between *C. calidirosea* isolates.** Tie-corrected Spearman's rho of redox dye responses to carbon sources (95 substrates including various carbohydrates, amino acids, and their derivatives) on the BIOLOG PM1 phenotype microarray plates (http://www.biolog.com/pdf/pm_lit/PM1-PM10.pdf), using means of duplicates of each isolate (pooled standard deviation of the spectroscopic observations: T49 - 0.0489, P488 - 0.0463, WRG1.2 - 0.0675, TKA4.10 - 0.0194). Overall, the isolates demonstrated high physiological similarity in the utilization of carbohydrates. However, differences in Spearman's rank order correlation between isolates were still observed. Analysis of the range of rank-order correlations showed P488 and WRG1.2 with the highest correlation (0.94), and T49$^T$ and WRG1.2 with the lowest redox response profile correlation (0.77) in carbon source utilization.

# 8 Soil Mineral Analysis

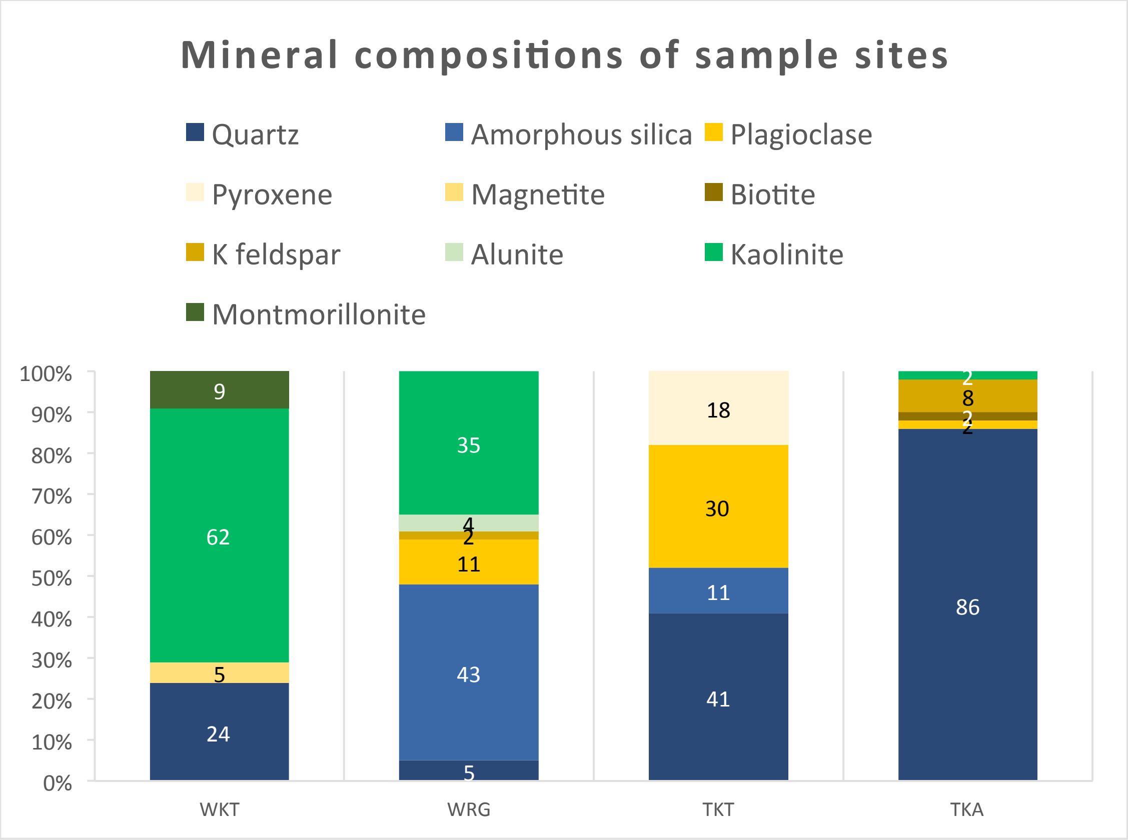


**Figure S7 - Mineral composition of sample sites**. The mineral compositions of each sample site were determined by quantitative X-ray diffraction analysis. The Y-axis indicates the percentage of total composition. Yellow hues indicate primary minerals which are sensitive to hydrothermal alteration. Green hues indicate secondary clay minerals resulting from hydrothermal alteration. Blue hues indicate quartz and amorphous silica, which are non-clay minerals from which the effects of hydrothermal alteration could not be directly determined. The samples sites are arranged in descending order of relative abundance of clay minerals.

# 9 Community 16S rRNA gene-targeted sequencing and processing

Total soil DNA was extracted with a NucleoSpin Soil DNA kit (Macherey-Nagel GmbH & Co. KG, Düren, Germany). Each soil sample was mixed with 500 µL of 50 g/L sterile skim milk solution (Becton, Dickinson and Company, NJ, USA) to reduce DNA binding to clay soil before extraction as per the manufacturer's instructions. Fusion primers were constructed to amplify a 350-bp amplicon on the V4 16S rRNA gene region, using the "universal" prokaryotic primers f515 and r806 (56). The forward fusion primer consisted of the "A" adaptor, an IonXpress barcode sequence, a GAT barcode adaptor and the f515 primer. The reverse fusion primer contained the P1 sequence and r806 primer (57). PCR reactions were performed in 15 µL reactions, each containing 0.304 µL (10 µM concentration) of forward and reverse primers, 0.61 µL of BSA (10 mg/mL), 1.83 µL of dNTPs (8 mM), 1.83 µL of 10X PCR buffer, 1.83 µL of $MgCl_2$ (50 mM), and 1 µL of soil community DNA as a template. Prior to the addition of primers and template, the PCR master mix was treated with 0.53 µL of ethidium monoazide bromide (1/200 dilution from 1 mg/mL stock) per reaction, and with 1 min of photoactivation using a lamp to remove trace DNA contaminants (58). The PCR reagents, including ethidium monoazide bromide, were supplied by Invitrogen (Invitrogen, Carlsbad, CA, USA). PCR was conducted using the following thermocycling parameters after the initial 3 min of 94 °C denaturation: 30 cycles (94 °C, 45 sec; 50 °C, 1 min; 72 °C, 1.5 min), followed by a final 10 min incubation at 72 °C. The amplicons from triplicate PCR products were pooled and purified using SPRIselect (Beckman Coulter Inc.) to remove small nucleic acid fragments (left-hand selection). Amplicon concentration and quality were verified and adjusted to 26 pM with a Qubit fluorimeter (high-sensitivity) (Life Technologies) and a 9100 BioAnalyser (Agilent Technologies) prior to pooling amplicons from all samples together for emulsion PCR. DNA sequencing was conducted using Ion Torrent PGM (Life Technologies) with an Ion 318v2 chip, using 400 bp chemistry, and generated ~217,000 raw sequencing reads.

UPARSE (59) was used to process 216,944 raw sequencing reads, with the following workflow to remove anomalous sequences. First, reads with lengths outside 276-344 bp range were removed. Next, the forward primer and barcode segments of the reads were trimmed off, and the resulting reads were subjected to Q score filtering to remove reads with maximum expected error >3. Singleton reads were then removed, and the sequences were globally trimmed to 250 bp. A total of 85,063 high quality sequences were obtained after quality control from the four sample sites, with a mean of 20,733 sequences and a range of 17,941 to 25,656 sequences per sample. Rarefaction curves for the sample sites are shown in (**Figure S8**).

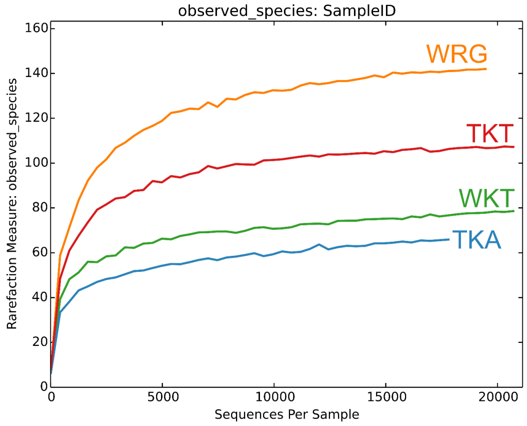


**Figure S8 -** Observed OTUs in the four sample sites. Rarefaction curves of observed taxa (as defined by OTU clustering with 97 % criterion) in the four sample site communities showing plateauing of observed taxa with increased numbers of sequences sampled, thus indicating a majority of targeted biodiversity were represented.

## 10 Map of the Taupō Volcanic Zone (TVZ)

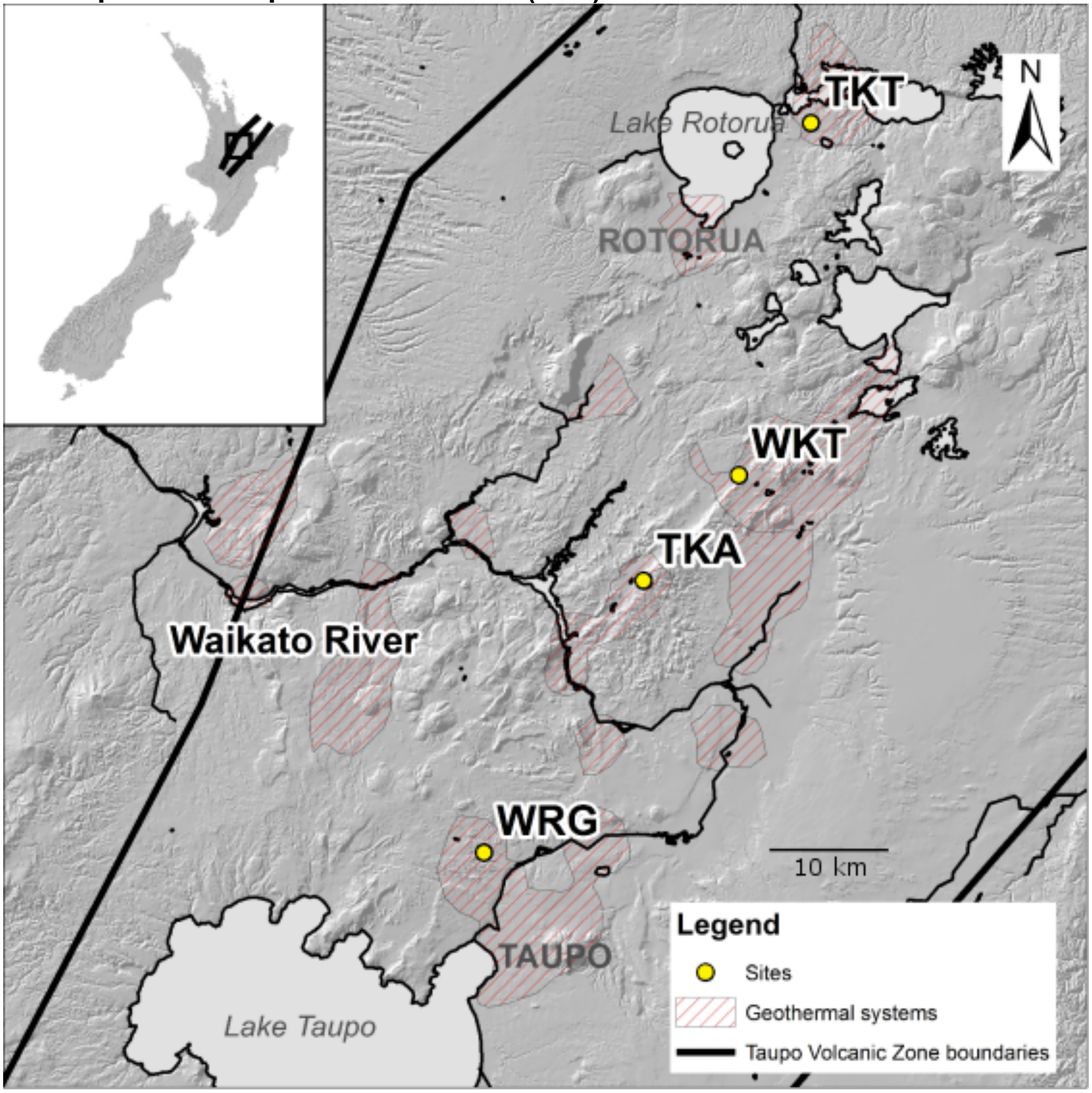


**Figure S9 - Map of the Taupō Volcanic Zone (TVZ).** GIS layers indicate geothermal fields, digital topography, and rivers and streams. The sample sites are indicated on the map. Wairakei – WRG, Te Kopia – TKA, Waikite – WKT, Tikitere – TKT. Pairwise distances between sites: TKT-WKT = 30.3 km, TKT-TKA = 41.0 km, TKT-WRG = 67.4 km, WKT-TKA = 12.0 km, WKT-WRG = 38.4 km, and TKA-WRG = 26.6 km. The scale bar indicates the distance of 10 km.

# 11 Putative HGT regions

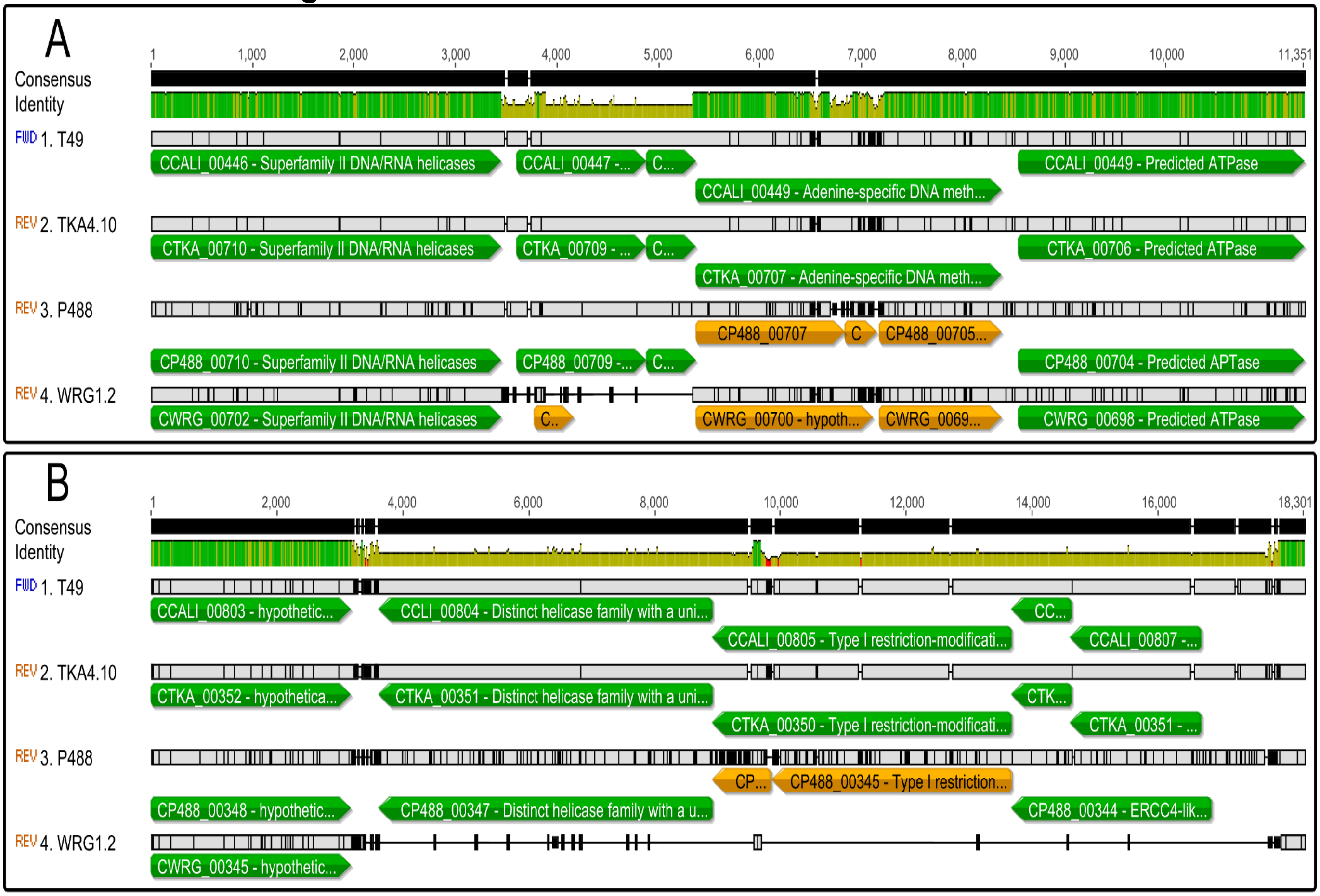


**Figure S10 - Alignments of putative HGT regions.** Multiple MUSCLE (60) alignments of putative HGT regions of *C. calidirosea* isolate (T49$^{T}$, TKA4.10, P488, WRG1.2) genomes, relative to two T49$^{T}$ loci: (A) CCALI_00447-00449 and (B) CCALI_00804-00807. Black vertical boxes indicate regions of disagreement, and horizontal line indicate gaps in alignments. P488 and WRG1.2 contain regions that are affected by many deletions and therefore likely to contain non-functional pseudogenes, which are indicated by yellow segments (putative DNA methylase, repair protein, and helicase).

## References


1. **Benson DA**, **Karsch-Mizrachi I**, **Lipman DJ**, **Ostell J**, **Sayers EW**. 2011. GenBank. Nucleic Acids Res. **39**:D32–7.

2. **Gilbert JA**, **Bailey M**, **Field D**, **Fierer N**, **Fuhrman JA**, **Hu B**, **Jansson J**, **Knight R**, **Kowalchuk GA**, **Kyrpides NC**, **Meyer F**, **Stevens R**. 2011. The Earth Microbiome Project: The Meeting Report for the 1st International Earth Microbiome Project Conference, Shenzhen, China, June 13th-15th 2011. Stand. Genomic Sci. **5**:243–247.

3. **Somers JM**, **Amzallag A**, **Middleton RB**. 1973. Genetic Fine Structure of the Leucine Operon of *Escherichia coli* K-12. J. Bacteriol. **113**:1268–1272.

4. **Goldschmidt EP**, **Cater MS**, **Matney TS**, **Butler MA**, **Greene A**. 1970. Genetic analysis of the histidine operon in *Escherichia coli* K12. Genetics **66**:219–29.

5. **Zalkin H**, **Ebbole DJ**. 1988. Organization and regulation of genes encoding biosynthetic enzymes in *Bacillus subtilis*. J. Biol. Chem. **263**:1595–1598.

6. **Roth CW**, **Nester EW**. 1971. Co-ordinate control of tryptophan, histidine and tyrosine enzyme synthesis in *Bacillus subtilis*. J. Mol. Biol. **62**:577–589.

7. **Crawford IP**. 1980. Comparative studies on the regulation of tryptophan synthesis. CRC Crit. Rev. Biochem. **8**:175–89.

8. **Markowitz VM**, **Chen I-MA**, **Palaniappan K**, **Chu K**, **Szeto E**, **Grechkin Y**, **Ratner A**, **Jacob B**, **Huang J**, **Williams P**, **Huntemann M**, **Anderson I**, **Mavromatis K**, **Ivanova NN**, **Kyrpides NC**. 2012. IMG: the Integrated Microbial Genomes database and comparative analysis system. Nucleic Acids Res. **40**:D115–22.

9. **Wickham H**. 2009. ggplot2: elegant graphics for data analysis. Springer New York, New York, NY.

10. **R Core Team**. 2013. R: A language and environment for statistical computing. Vienna, Austria.

11. **Letunic I**, **Bork P**. 2007. Interactive Tree Of Life (iTOL): an online tool for phylogenetic tree display and annotation. Bioinformatics **23**:127–8.

12. **Lee KC**, **Morgan XC**, **Dunfield PF**, **Tamas I**, **McDonald IR**, **Stott MB**. 2014. Genomic analysis of *Chthonomonas calidirosea*, the first sequenced isolate of the phylum Armatimonadetes. ISME J. **8**:1522–1533.

13. **Hayashi K**, **Morooka N**, **Yamamoto Y**, **Fujita K**, **Isono K**, **Choi S**, **Ohtsubo E**, **Baba T**, **Wanner BL**, **Mori H**, **Horiuchi T**. 2006. Highly accurate genome sequences of *Escherichia coli* K-12 strains MG1655 and W3110. Mol. Syst. Biol. **2**:2006.0007.

14. **Jeong H**, **Barbe V**, **Lee CH**, **Vallenet D**, **Yu DS**, **Choi S-H**, **Couloux A**, **Lee S-W**, **Yoon SH**, **Cattolico L**, **Hur C-G**,

**Park H-S**, **Ségurens B**, **Kim SC**, **Oh TK**, **Lenski RE**, **Studier FW**, **Daegelen P**, **Kim JF**. 2009. Genome sequences of *Escherichia col*i B strains REL606 and BL21(DE3). J. Mol. Biol. **394**:644–52.

15. **Yang F**, **Yang J**, **Zhang X**, **Chen L**, **Jiang Y**, **Yan Y**, **Tang X**, **Wang J**, **Xiong Z**, **Dong J**, **Xue Y**, **Zhu Y**, **Xu X**, **Sun L**, **Chen S**, **Nie H**, **Peng J**, **Xu J**, **Wang Y**, **Yuan Z**, **Wen Y**, **Yao Z**, **Shen Y**, **Qiang B**, **Hou Y**, **Yu J**, **Jin Q**. 2005. Genome dynamics and diversity of *Shigella* species, the etiologic agents of bacillary dysentery. Nucleic Acids Res. **33**:6445–58.

16. **Stover CK**, **Pham XQ**, **Erwin AL**, **Mizoguchi SD**, **Warrener P**, **Hickey MJ**, **Brinkman FS**, **Hufnagle WO**, **Kowalik DJ**, **Lagrou M**, **Garber RL**, **Goltry L**, **Tolentino E**, **Westbrock-Wadman S**, **Yuan Y**, **Brody LL**, **Coulter SN**, **Folger KR**, **Kas A**, **Larbig K**, **Lim R**, **Smith K**, **Spencer D**, **Wong GK**, **Wu Z**, **Paulsen IT**, **Reizer J**, **Saier MH**, **Hancock RE**, **Lory S**, **Olson M V**. 2000. Complete genome sequence of *Pseudomonas aeruginosa* PAO1, an opportunistic pathogen. Nature **406**:959–64.

17. **Badger JH**, **Hoover TR**, **Brun Y V**, **Weiner RM**, **Laub MT**, **Alexandre G**, **Mrázek J**, **Ren Q**, **Paulsen IT**, **Nelson KE**, **Khouri HM**, **Radune D**, **Sosa J**, **Dodson RJ**, **Sullivan SA**, **Rosovitz MJ**, **Madupu R**, **Brinkac LM**, **Durkin AS**, **Daugherty SC**, **Kothari SP**, **Giglio MG**, **Zhou L**, **Haft DH**, **Selengut JD**, **Davidsen TM**, **Yang Q**, **Zafar N**, **Ward NL**. 2006. Comparative genomic evidence for a close relationship between the dimorphic prosthecate bacteria *Hyphomonas neptunium* and *Caulobacter crescentus*. J. Bacteriol. **188**:6841–50.

18. **Aklujkar M**, **Young ND**, **Holmes D**, **Chavan M**, **Risso C**, **Kiss HE**, **Han CS**, **Land ML**, **Lovley DR**. 2010. The genome of *Geobacter bemidjiensis*, exemplar for the subsurface clade of *Geobacter* species that predominate in Fe(III)-reducing subsurface environments. BMC Genomics **11**:490.

19. **Ivanova N**, **Rohde C**, **Munk C**, **Nolan M**, **Lucas S**, **Del Rio TG**, **Tice H**, **Deshpande S**, **Cheng J-F**, **Tapia R**, **Han C**, **Goodwin L**, **Pitluck S**, **Liolios K**, **Mavromatis K**, **Mikhailova N**, **Pati A**, **Chen A**, **Palaniappan K**, **Land M**, **Hauser L**, **Chang Y-J**, **Jeffries CD**, **Brambilla E**, **Rohde M**, **Göker M**, **Tindall BJ**, **Woyke T**, **Bristow J**, **Eisen JA**, **Markowitz V**, **Hugenholtz P**, **Kyrpides NC**, **Klenk H-P**, **Lapidus A**. 2011. Complete genome sequence of *Truepera radiovictrix* type strain (RQ-24). Stand. Genomic Sci. **4**:91–9.

20. **Tindall BJ**, **Sikorski J**, **Lucas S**, **Goltsman E**, **Copeland A**, **Glavina Del Rio T**, **Nolan M**, **Tice H**, **Cheng J-F**, **Han C**, **Pitluck S**, **Liolios K**, **Ivanova N**, **Mavromatis K**, **Ovchinnikova G**, **Pati A**, **Fähnrich R**, **Goodwin L**, **Chen A**, **Palaniappan K**, **Land M**, **Hauser L**, **Chang Y-J**, **Jeffries CD**, **Rohde M**, **Göker M**, **Woyke T**, **Bristow J**, **Eisen JA**, **Markowitz V**, **Hugenholtz P**, **Kyrpides NC**, **Klenk H-P**, **Lapidus A**. 2010. Complete genome sequence of *Meiothermus ruber* type strain (21). Stand. Genomic Sci. **3**:26–36.

21. **Fujisawa T**, **Narikawa R**, **Okamoto S**, **Ehira S**, **Yoshimura H**, **Suzuki I**, **Masuda T**, **Mochimaru M**, **Takaichi S**, **Awai K**, **Sekine M**, **Horikawa H**, **Yashiro I**, **Omata S**, **Takarada H**, **Katano Y**, **Kosugi H**, **Tanikawa S**, **Ohmori K**, **Sato N**, **Ikeuchi M**, **Fujita N**, **Ohmori M**. 2010. Genomic structure of an economically important cyanobacterium, *Arthrospira* (*Spirulina*) *platensis* NIES-39. DNA Res. **17**:85–103.

22. **Kettler GC**, **Martiny AC**, **Huang K**, **Zucker J**, **Coleman ML**, **Rodrigue S**, **Chen F**, **Lapidus A**, **Ferriera S**, **Johnson J**, **Steglich C**, **Church GM**, **Richardson P**, **Chisholm SW**. 2007. Patterns and implications of gene gain and loss in the evolution of *Prochlorococcus*. PLoS Genet. **3**:e231.

23. **Hu Z-Y**, **Wang Y-Z**, **Im W-T**, **Wang S-Y**, **Zhao G-P**, **Zheng H-J**, **Quan Z-X**. 2014. The first complete genome sequence of the Class *Fimbriimonadia* in the Phylum *Armatimonadetes*. PLoS One **9**:e100794.

24. **Tang K-H**, **Barry K**, **Chertkov O**, **Dalin E**, **Han CS**, **Hauser LJ**, **Honchak BM**, **Karbach LE**, **Land ML**, **Lapidus A**, **Larimer FW**, **Mikhailova N**, **Pitluck S**, **Pierson BK**, **Blankenship RE**. 2011. Complete genome sequence of the filamentous anoxygenic phototrophic bacterium *Chloroflexus aurantiacus*. BMC Genomics **12**:334.

25. **Kube M**, **Beck A**, **Zinder SH**, **Kuhl H**, **Reinhardt R**, **Adrian L**. 2005. Genome sequence of the chlorinated compound-respiring bacterium *Dehalococcoides* species strain CBDB1. Nat. Biotechnol. **23**:1269–73.

26. **Latif H**, **Lerman JA**, **Portnoy VA**, **Tarasova Y**, **Nagarajan H**, **Schrimpe-Rutledge AC**, **Smith RD**, **Adkins JN**, **Lee D-H**, **Qiu Y**, **Zengler K**. 2013. The genome organization of *Thermotoga maritima* reflects its lifestyle. PLoS Genet. **9**:e1003485.

27. **Barabote RD**, **Xie G**, **Leu DH**, **Normand P**, **Necsulea A**, **Daubin V**, **Médigue C**, **Adney WS**, **Xu XC**, **Lapidus A**, **Parales RE**, **Detter C**, **Pujic P**, **Bruce D**, **Lavire C**, **Challacombe JF**, **Brettin TS**, **Berry AM**. 2009. Complete genome of the cellulolytic thermophile *Acidothermus cellulolyticus* 11B provides insights into its ecophysiological and evolutionary adaptations. Genome Res. **19**:1033–43.

28. **Garnier T**, **Eiglmeier K**, **Camus J-C**, **Medina N**, **Mansoor H**, **Pryor M**, **Duthoy S**, **Grondin S**, **Lacroix C**, **Monsempe C**, **Simon S**, **Harris B**, **Atkin R**, **Doggett J**, **Mayes R**, **Keating L**, **Wheeler PR**, **Parkhill J**, **Barrell BG**, **Cole ST**, **Gordon S V**, **Hewinson RG**. 2003. The complete genome sequence of *Mycobacterium bovis*. Proc. Natl. Acad. Sci. U. S. A. **100**:7877–82.

29. **Seki M**, **Honda I**, **Fujita I**, **Yano I**, **Yamamoto S**, **Koyama A**. 2009. Whole genome sequence analysis of *Mycobacterium bovis* bacillus Calmette-Guérin (BCG) Tokyo 172: a comparative study of BCG vaccine substrains. Vaccine **27**:1710–6.

30. **van de Werken HJG**, **Verhaart MRA**, **VanFossen AL**, **Willquist K**, **Lewis DL**, **Nichols JD**, **Goorissen HP**, **Mongodin EF**, **Nelson KE**, **van Niel EWJ**, **Stams AJM**, **Ward DE**, **de Vos WM**, **van der Oost J**, **Kelly RM**, **Kengen SWM**. 2008. Hydrogenomics of the extremely thermophilic bacterium *Caldicellulosiruptor saccharolyticus*. Appl. Environ. Microbiol. **74**:6720–9.

31. **Wilson CM**, **Rodriguez M**, **Johnson CM**, **Martin SL**, **Chu TM**, **Wolfinger RD**, **Hauser LJ**, **Land ML**, **Klingeman DM**, **Syed MH**, **Ragauskas AJ**, **Tschaplinski TJ**, **Mielenz JR**, **Brown SD**. 2013. Global transcriptome analysis of *Clostridium thermocellum* ATCC 27405 during growth on dilute acid pretreated Populus and switchgrass. Biotechnol. Biofuels **6**:179.

32. **Kunst F**, **Ogasawara N**, **Moszer I**, **Albertini AM**, **Alloni G**, **Azevedo V**, **Bertero MG**, **Bessières P**, **Bolotin A**, **Borchert S**, **Borriss R**, **Boursier L**, **Brans A**, **Braun M**, **Brignell SC**, **Bron S**, **Brouillet S**, **Bruschi C V**, **Caldwell B**, **Capuano V**, **Carter NM**, **Choi SK**, **Cordani JJ**, **Connerton IF**, **Cummings NJ**, **Daniel RA**, **Denziot F**, **Devine KM**, **Düsterhöft A**, **Ehrlich SD**, **Emmerson PT**, **Entian KD**, **Errington J**, **Fabret C**, **Ferrari E**, **Foulger D**, **Fritz C**, **Fujita M**, **Fujita Y**, **Fuma S**, **Galizzi A**, **Galleron N**, **Ghim SY**, **Glaser P**, **Goffeau A**, **Golightly EJ**, **Grandi G**, **Guiseppi G**, **Guy BJ**, **Haga K**, **Haiech J**, **Harwood CR**, **Hènaut A**, **Hilbert H**, **Holsappel S**, **Hosono S**, **Hullo MF**, **Itaya M**, **Jones L**, **Joris B**, **Karamata D**, **Kasahara Y**, **Klaerr-Blanchard M**, **Klein C**, **Kobayashi Y**, **Koetter P**, **Koningstein G**, **Krogh S**, **Kumano M**, **Kurita K**, **Lapidus A**, **Lardinois S**, **Lauber J**, **Lazarevic V**, **Lee SM**, **Levine A**, **Liu H**, **Masuda S**, **Mauël C**, **Médigue C**, **Medina N**, **Mellado RP**, **Mizuno M**, **Moestl D**, **Nakai S**, **Noback M**, **Noone D**, **O'Reilly M**, **Ogawa K**, **Ogiwara A**, **Oudega B**, **Park SH**, **Parro V**, **Pohl TM**, **Portelle D**, **Porwollik S**, **Prescott AM**, **Presecan E**, **Pujic P**, **Purnelle B**, **Rapoport G**, **Rey M**, **Reynolds S**, **Rieger M**, **Rivolta C**, **Rocha E**, **Roche B**, **Rose M**, **Sadaie Y**, **Sato T**, **Scanlan E**, **Schleich S**, **Schroeter R**, **Scoffone F**, **Sekiguchi J**, **Sekowska A**, **Seror SJ**, **Serror P**, **Shin BS**, **Soldo B**, **Sorokin A**, **Tacconi E**, **Takagi T**, **Takahashi H**, **Takemaru K**,

**Takeuchi M**, **Tamakoshi A**, **Tanaka T**, **Terpstra P**, **Togoni A**, **Tosato V**, **Uchiyama S**, **Vandebol M**, **Vannier F**, **Vassarotti A**, **Viari A**, **Wambutt R**, **Wedler H**, **Weitzenegger T**, **Winters P**, **Wipat A**, **Yamamoto H**, **Yamane K**, **Yasumoto K**, **Yata K**, **Yoshida K**, **Yoshikawa HF**, **Zumstein E**, **Yoshikawa H**, **Danchin A**. 1997. The complete genome sequence of the gram-positive bacterium *Bacillus subtilis*. Nature **390**:249–56.

33. **Kabisch J**, **Thürmer A**, **Hübel T**, **Popper L**, **Daniel R**, **Schweder T**. 2013. Characterization and optimization of *Bacillus subtilis* ATCC 6051 as an expression host. J. Biotechnol. **163**:97–104.

34. **Lawrence J**. 1999. Selfish operons: the evolutionary impact of gene clustering in prokaryotes and eukaryotes. Curr. Opin. Genet. Dev. **9**:642–8.

35. **Martin FJ**, **McInerney JO**. 2009. Recurring cluster and operon assembly for Phenylacetate degradation genes. BMC Evol. Biol. **9**:36.

36. **Ballouz S**, **Francis AR**, **Lan R**, **Tanaka MM**. 2010. Conditions for the evolution of gene clusters in bacterial genomes. PLoS Comput. Biol. **6**:e1000672.

37. **Yanai I**, **Mellor JC**, **DeLisi C**. 2002. Identifying functional links between genes using conserved chromosomal proximity. Trends Genet. **18**:176–9.

38. **Overbeek R**, **Fonstein M**, **D'Souza M**, **Pusch GD**, **Maltsev N**. 1999. The use of gene clusters to infer functional coupling. Proc. Natl. Acad. Sci. U. S. A. **96**:2896–901.

39. **Wang B**, **Lu L**, **Lv H**, **Jiang H**, **Qu G**, **Tian C**, **Ma Y**. 2014. The transcriptome landscape of Prochlorococcus MED4 and the factors for stabilizing the core genome. BMC Microbiol. **14**:11.

40. **Zheng Y**, **Szustakowski JD**, **Fortnow L**, **Roberts RJ**, **Kasif S**. 2002. Computational identification of operons in microbial genomes. Genome Res. **12**:1221–30.

41. **Xie G**, **Keyhani NO**, **Bonner CA**, **Jensen RA**. 2003. Ancient origin of the tryptophan operon and the dynamics of evolutionary change. Microbiol. Mol. Biol. Rev. **67**:303–42, table of contents.

42. **Salgado H**, **Moreno-Hagelsieb G**, **Smith TF**, **Collado-Vides J**. 2000. Operons in *Escherichia coli*: Genomic analyses and predictions. Proc. Natl. Acad. Sci. **97**:6652–6657.

43. **Lathe WC**, **Snel B**, **Bork P**. 2000. Gene context conservation of a higher order than operons. Trends Biochem. Sci. **25**:474–9.

44. **Price MN**, **Huang KH**, **Arkin AP**, **Alm EJ**. 2005. Operon formation is driven by co-regulation and not by horizontal gene transfer. Genome Res. **15**:809–19.

45. **Galardini M**, **Biondi EG**, **Bazzicalupo M**, **Mengoni A**. 2011. CONTIGuator: a bacterial genomes finishing tool for structural insights on draft genomes. Source Code Biol. Med. **6**:11.

46. **Langmead B**, **Salzberg SL**. 2012. Fast gapped-read alignment with Bowtie 2. Nat. Methods **9**:357–9.

47. **Quinlan AR**. 2014. BEDTools: The Swiss-Army Tool for Genome Feature Analysis. Curr. Protoc. Bioinformatics **47**:11.12.1–11.12.34.

48. **Reno ML**, **Held NL**, **Fields CJ**, **Burke P V**, **Whitaker RJ**. 2009. Biogeography of the *Sulfolobus islandicus* pan-genome. Proc. Natl. Acad. Sci. U. S. A. **106**:8605–10.

49. **Oshima M**, **Ariga T**. 1975. Omega-cyclohexyl fatty acids in acidophilic thermophilic bacteria. Studies on their presence, structure, and biosynthesis using precursors labeled with stable isotopes and radioisotopes. J. Biol. Chem. **250**:6963–6968.

50. **Henne A**, **Brüggemann H**, **Raasch C**, **Wiezer A**, **Hartsch T**, **Liesegang H**, **Johann A**, **Lienard T**, **Gohl O**, **Martinez-Arias R**, **Jacobi C**, **Starkuviene V**, **Schlenczeck S**, **Dencker S**, **Huber R**, **Klenk H-P**, **Kramer W**, **Merkl R**, **Gottschalk G**, **Fritz H-J**. 2004. The genome sequence of the extreme thermophile *Thermus thermophilus*. Nat. Biotechnol. **22**:547–53.

51. **Jiang L**, **Lin M**, **Li X**, **Cui H**, **Xu X**, **Li S**, **Huang H**. 2013. Genome Sequence of *Thermus thermophilus* ATCC 33923, a thermostable trehalose-producing strain. Genome Announc. **1**.

52. **Oshima T**, **Imahori K**. 1971. Isolation of an extreme thermophile and thermostability of its transfer ribonucleic acid and ribosomes. J. Gen. Appl. Microbiol. **17**:513–517.

53. **Costa KC**, **Navarro JB**, **Shock EL**, **Zhang CL**, **Soukup D**, **Hedlund BP**. 2009. Microbiology and geochemistry of great boiling and mud hot springs in the United States Great Basin. Extremophiles **13**:447–59.

54. **Darling AE**, **Mau B**, **Perna NT**. 2010. progressiveMauve: multiple genome alignment with gene gain, loss and rearrangement. PLoS One **5**:e11147.

55. **Chevreux B**, **Wetter T**, **Suhai S**. 1999. Genome sequence assembly using trace signals and additional sequence information., p. 45–56. *In* German Conference on Bioinformatics.

56. **Caporaso JG**, **Lauber CL**, **Walters WA**, **Berg-Lyons D**, **Huntley J**, **Fierer N**, **Owens SM**, **Betley J**, **Fraser L**, **Bauer M**, **Gormley N**, **Gilbert JA**, **Smith G**, **Knight R**. 2012. Ultra-high-throughput microbial community analysis on the Illumina HiSeq and MiSeq platforms. ISME J. **6**:1621–4.

57. **Myllykangas S**, **Buenrostro J**, **Ji HP**. 2012. Bioinformatics for high throughput sequencing, p. 11–25. *In* Rodríguez-Ezpeleta, N, Hackenberg, M, Aransay, AM (eds.), . Springer New York, New York, NY.

58. **Rueckert A**, **Morgan HW**. 2007. Removal of contaminating DNA from polymerase chain reaction using ethidium monoazide. J. Microbiol. Methods **68**:596–600.

59. **Edgar RC**. 2013. UPARSE: highly accurate OTU sequences from microbial amplicon reads. Nat. Methods **10**:996–8.

60. **Edgar RC**. 2004. MUSCLE: multiple sequence alignment with high accuracy and high throughput. Nucleic

Acids Res. **32**:1792–7.
